# Lactylation of USP11 fosters breast cancer development by stabilizing CDK4 and activating CDK4-RB1-E2F1 pathway

**DOI:** 10.1038/s41419-026-09172-4

**Published:** 2026-08-12

**Authors:** Chao Wang, Siru Zhou, Yulin Li, Yalin Liang, Yuxin Shi, Dongyue Zhu, Xiaofeng Zheng

**Affiliations:** 1 State Key Laboratory of Gene Function and Modulation Research, School of Life Sciences, Peking University, Beijing, China; 2 Department of Biochemistry and Molecular Biology, School of Life Sciences, Peking University, 100871 Beijing, China

**Keywords:** Ubiquitylation, Breast cancer

## Abstract

Cyclin-dependent kinase 4 (CDK4) is a key player in the cell cycle. Its frequent overexpression in a multitude of cancers correlates with increased cell proliferation and resistance to CDK4 inhibitors, highlighting the critical need to target CDK4 degradation as a therapeutic approach. However, the mechanisms governing the maintenance and homeostasis of CDK4 protein levels remain elusive. Here, we identify that deubiquitinase USP11 promotes breast cancer progression by stabilizing CDK4. Mechanistically, we demonstrate that USP11 directly interacts with CDK4 and specifically targets the Lys48-linked polyubiquitination chains at K88, K106, and K142 sites in a DUB activity-dependent manner. USP11 knockout significantly reduces CDK4 abundance, consequently inhibiting the CDK4-RB1-E2F1 pathway, cell cycle progression, and proliferation. Warburg effect-generated lactate induces lactylation of USP11 at K385 and K766 sites, which enhances its binding to CDK4. High USP11 and CDK4 expressions in breast cancer correlates with tumor progression and poor prognosis. Moreover, the co-administration of CDK4 and USP11 inhibitors exhibits synergistic efficacy in breast cancer treatment of syngeneic mouse model. This research elucidates the role of USP11 in breast cancer through the deubiquitination and stabilization of CDK4, suggesting a dual inhibitor approach as a potential therapeutic strategy.

## Introduction

Cyclin-dependent kinase 4 (CDK4) plays a vital role in cell cycle [1]. During the mid to late G1 phase, the activated Cyclin D-CDK4/6 complex phosphorylates Retinoblastoma protein 1 (RB1), leading to the release of the transcription factor E2F1, which then promotes the expression of genes essential for cell cycle progression [2]. CDK4/6-RB1-E2F1 pathway integrates extracellular and intracellular signals and decides whether to trigger cell proliferation [3–5]. Given that cancer cells achieve uncontrolled proliferation by sustaining mitogenic signals and evading growth suppression [6], three ATP-competitive CDK4/6 inhibitors, Palbociclib, Ribociclib, and Abemaciclib, have been developed and applied in clinical cancer treatment [7–10].

Although CDK4 protein levels are stable during the cell cycle [11], its overexpression is a common feature in various cancers and is sufficient to induce tumorigenesis in mice [12–15]. Conversely, knockdown of CDK4 or CDK6 in mice reduces tumor susceptibility [16–18]. Moreover, the usage of CDK4 inhibitors is limited in Triple-Negative Breast Cancer (TNBC), and elevated CDK4 levels are associated with resistance to CDK4 inhibitors [19–21]. Thus, targeting the protein levels of CDK4 could provide new solutions to combat cancer and overcome drug resistance. Recent research reported that SKP2 promotes the proteasomal degradation of CDK4 while PAQR4 enhances CDK4 stability [22]. However, the specific deubiquitinating enzymes (DUBs) responsible for CDK4 stabilization remain to be identified.

The ubiquitin-proteasome system mediates the degradation of nearly 80% of proteins. The process of ubiquitination is counterbalanced by deubiquitinating enzymes. Ubiquitin carboxyl-terminal hydrolase 11 (USP11, also known as UHX1), located on the X chromosome, is a member of the USP subfamily of DUBs. It plays a pivotal role in the DNA damage repair by facilitating the formation of the RAD51-BRCA1-BRCA2 complex [23]. USP11 is recruited to DNA damage sites, where it deubiquitinates histones H2A K119 and H2B K120 [24]. It also regulates nuclear protein transport by deubiquitinating Ran-BPM, thereby preventing protein degradation and promoting nuclear import [25]. In signaling pathways, USP11 inhibits NF-κB activation through deubiquitination of IκBα [26]. In the TGF-β pathway, USP11 enhances signal transduction by deubiquitinating both ALK5 and TGFBR2. The stabilization of TGFBR2 in particular drives epithelial–mesenchymal transition (EMT) and promotes tumor metastasis [27, 28]. Additionally, USP11 suppresses apoptosis by deubiquitinating cIAP2 and XIAP, enabling cells to resist apoptotic signals [29, 30].

Recent studies have revealed a complex and context-dependent role for USP11 in cancer. In lung cancer and colorectal cancer cells, USP11 stabilizes the CDK inhibitor p21 to induce cell cycle arrest [31]. Conversely, in hepatocellular carcinoma cells, it stabilizes the transcription factor E2F1 in the RB1 pathway to promote proliferation [32]. Specifically in breast cancer, USP11 has been reported to both suppress tumorigenesis by stabilizing PTEN [33] and promote malignancy by stabilizing XIAP and TGFBR2, as well as regulating ERα activity [28, 30, 34]. This functional versatility suggests that the substrate selectivity and biological functions of USP11 are highly dependent on cell type, tumor-driving pathways, and microenvironmental signals.

Lactylation (also known as lysine lactylation, Kla) is a recently identified post-translational modification. Here, we demonstrate that the lactylation of USP11 promotes the proliferation and progression of breast cancer by stabilizing CDK4 protein levels and facilitating the activation of CDK4-RB1-E2F1 signaling pathway. Consequently, synergistic inhibition of USP11 and CDK4 potently delivers a “second hit” to this pathway, leading to significantly enhanced tumor cell cytotoxicity. Our research not only highlights the pivotal link between CDK4 ubiquitination and tumor development, but also offers a dual-inhibitor strategy as a promising therapeutic intervention.

## Results

### Deubiquitinase USP11 directly interacts with CDK4

**Fig. 1:**
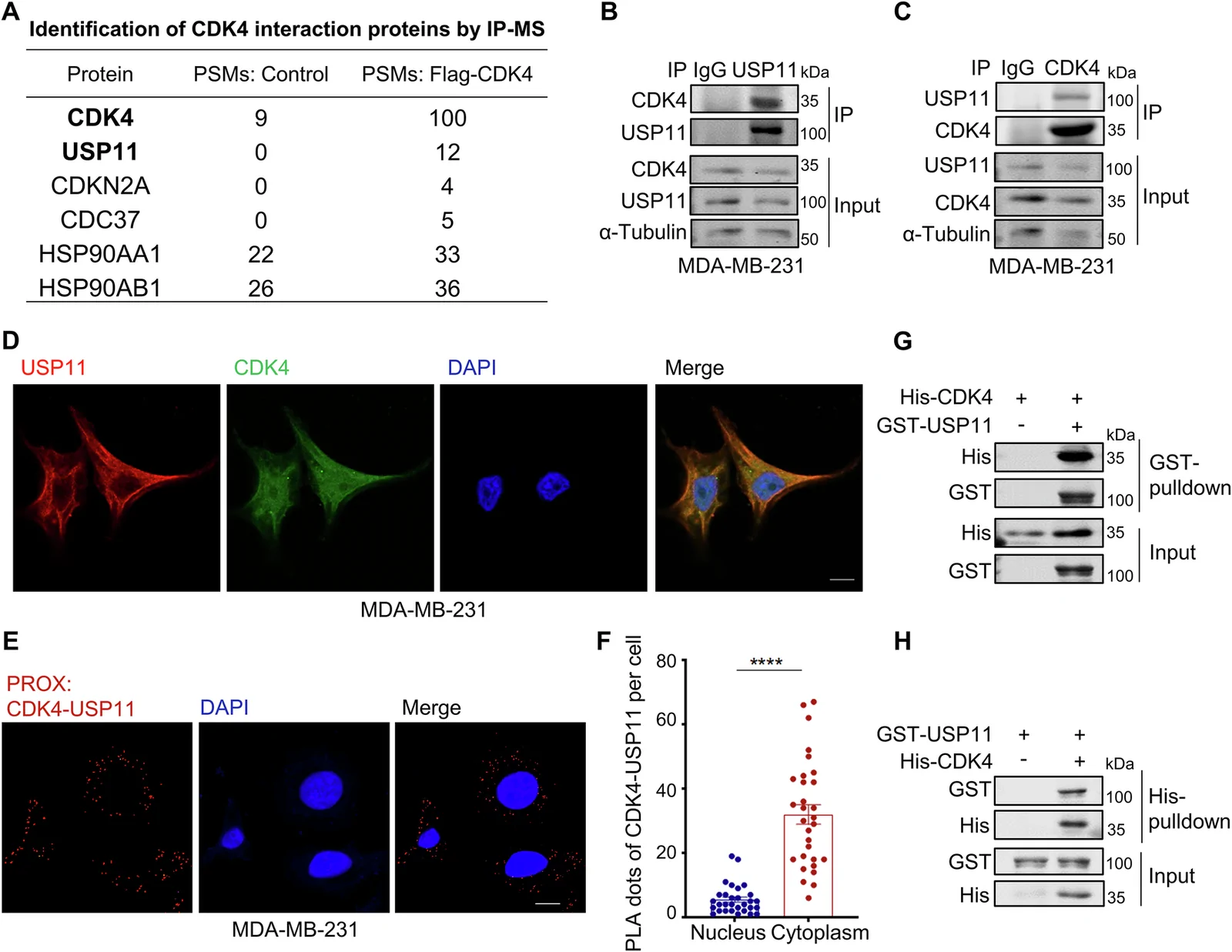
Deubiquitinase USP11 directly interacts with CDK4. **A** USP11 was identified among the CDK4 interactors through mass spectrometry analysis of Flag-USP11-overexpressing HEK-293T cells. **B**, **C** The endogenous interaction between USP11 and CDK4 was characterized in MDA-MB-231 cells. **D** Colocalization of CDK4 and USP11 was observed using an immunofluorescence assay. Scale bar, 10 μm. **E** The in situ interaction between CDK4 and USP11 was visualized by Duolink-PLA assay, revealing the interaction mainly occurred in the cytoplasm. Scale bar, 10 μm. **F** Quantitative analysis of PLA dots, indicative of USP11-CDK4 interaction, in the nucleus or cytoplasm is presented as mean ± SEM (*n* = 30). Statistical analysis was performed using a Student’s *t* test. *P < 0.05, **P < 0.01, ***P < 0.001, ****P < 0.001. **G**, **H** The direct interaction between CDK4 and USP11 was ascertained through an in vitro GST-pulldown assay using recombinant His-CDK4 and GST-USP11 proteins purified from *E. coli*.

Given that the overexpression of CDK4 in a variety of cancers is associated with enhanced cell proliferation and resistance to CDK4 inhibitors, modulating the protein levels of CDK4 may offer promising strategies to overcome drug resistance. In order to search for deubiquitinases of CDK4, we transfected HEK-293T cells with Flag-tagged CDK4 and subsequently performed selective immunoprecipitation using Flag M2-agarose beads. Mass spectrometry analysis of the precipitated proteins revealed the presence of USP11 among the enriched interactors of CDK4 (Fig. 1A), suggesting that USP11 may interact with CDK4. We confirmed the interaction between CDK4 and USP11 by co-immunoprecipitation (co-IP) in HEK-293T cells under both exogenous and endogenous conditions (Fig. S1A–C). This interaction was further validated in various breast cancer cell lines, including MDA-MB-231, MCF7, and T47D (Figs. 1B, C, S1D and S1E). Moreover, immunofluorescence assay revealed that CDK4 and USP11 colocalized predominantly in the cytoplasm (Fig. 1D). The in situ interaction was further characterized using the Duolink proximity ligation assay (PLA), which specifically confirmed that the CDK4-USP11 interaction mainly occurred in the cytoplasm (Fig. 1E, F). To assess the direct CDK4-USP11 interaction, we performed an in vitro pulldown assay with recombinant His-CDK4 and GST-USP11 proteins expressed in *E. coli* (Fig. 1G, H). These results demonstrate that deubiquitinase USP11 interacts directly with CDK4.

### USP11 cleaves the K48-linked polyubiquitination chains on CDK4 at lysine residues 88, 106, and 142 in a DUB activity-dependent manner

**Fig. 2:**
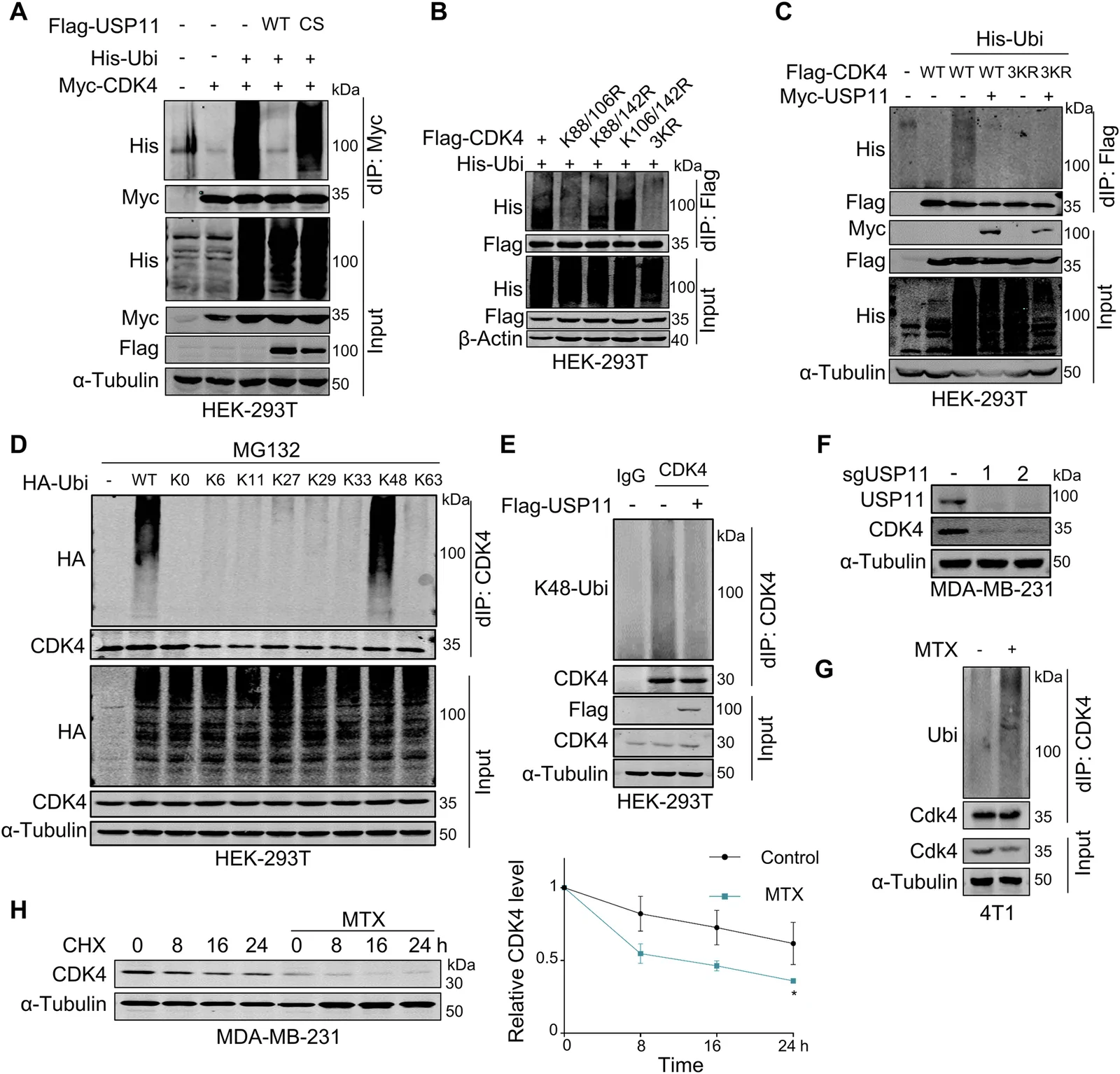
USP11 deubiquitinates and stabilizes CDK4. **A** The influence of USP11 on CDK4 ubiquitination was assessed using denaturing immunoprecipitation (dIP) assays. HEK-293T cells were transfected with the indicated plasmids for 48 h, after which the ubiquitination status of CDK4 was determined through dIP. USP11-C318S represents a mutant lacking deubiquitinating activity. **B** The specific ubiquitination sites of CDK4 were identified using dIP assays. HEK-293T cells were transfected with His-Ubi and Flag-CDK4-WT, along with various K-to-R mutant as indicated, for a duration of 48 h, and then the ubiquitination levels of CDK4 and the respective mutants were assessed. **C** The impact of USP11 on the ubiquitination of CDK4 at specific sites was investigated by dIP assays in HEK-293T cells that transfected with the relevant plasmids. **D** HA-Ubi and its K-only mutants were transfected into the HEK-293T cell line. After 48 h of culture, dIP was performed to detect the polyubiquitination of CDK4. **E** The effect of USP11 on the K48-linked polyubiquitination of endogenous CDK4 protein was detected by dIP assay. **F** USP11 knockout (KO) cell line of MDA-MB-231 was successfully established using the CRISPR-Cas9 system. **G** Inhibition of USP11 using MTX led to an increase in the ubiquitination of endogenous CDK4 in 4T1 cells. **H** The half-life of CDK4 protein was examined in MDA-MB-231 cells treated with 50 µg/mL CHX, under conditions with and without the addition of 5 nM MTX. The relative amount of CDK4 protein was quantified and normalized to the levels of α-Tubulin.

Given the direct interaction between CDK4 and USP11, we hypothesize that USP11 functions as its deubiquitinase. Indeed, USP11 overexpression significantly reduced CDK4 ubiquitination, supporting this hypothesis (Fig. 2A). To test whether USP11 regulates CDK4 in a DUB activity-dependent manner, we generated a catalytically inactive USP11-C318S mutant [35]. As expected, exogenous denaturing immunoprecipitation (dIP) demonstrated that wild type USP11, but not the USP11-C318S mutant, reduced CDK4 ubiquitination (Fig. 2A). Next, endogenous dIP assay also confirmed that wild type USP11 deubiquitinated CDK4 in both HEK-293T and MCF7 cells, whereas the USP11-C318S mutant had no such effect (Figs. S2A, S2B). Additionally, an in vitro deubiquitination assay proved that the ubiquitination of CDK4 was exclusively removed by the recombinant USP11 protein and not by the USP11-C318S mutant. Furthermore, the inhibition of USP11 by Mitoxantrone (MTX), an inhibitor of USP11, also failed to deubiquitinate CDK4 (Fig. S2C). These results indicate that USP11 removes the ubiquitination of CDK4 in a DUB activity-dependent manner.

Ubiquitination typically targets evolutionarily conserved lysine residues on the protein surface. CDK4 contains 11 lysine residues, all surface-exposed except K297 (structurally unresolved), and all are evolutionarily conserved (Figs. S2D, S2E). To identify functional ubiquitination sites, we systematically screened single lysine-to-arginine (K-to-R) mutations in HEK-293T cells. This revealed that K88, K106, and K142 are major ubiquitination sites, as their individual mutation reduced ubiquitination (Figs. S2F, S2G). We then generated double (K88/106 R, K88/142 R and K106/142 R) and triple (K88/106/142 R, CDK4-3KR) mutants. The CDK4-3KR mutant showed a pronounced decrease in ubiquitination (Fig. 2B), and notably, its ubiquitination level was no longer affected by USP11 overexpression (Fig. 2C). These findings identify lysines 88, 106, and 142 as the principal ubiquitination sites of CDK4.

Moreover, using K-only ubiquitin variants, we showed that CDK4 undergoes primarily K48-linked polyubiquitination (Fig. 2D). USP11 was shown to specifically remove these K48-linked chains from both overexpressed (Fig. S2H) and endogenous CDK4 (Fig. 2E). These findings indicate that USP11 deubiquitinates CDK4 by targeting its K48-linked polyubiquitination chains.

### USP11 stabilizes CDK4 protein

It is well established that K48-linked polyubiquitination directs proteins for degradation. We thus assessed whether USP11 stabilizes CDK4. We constructed USP11 knockout (KO) cell lines in 4T1, MCF7, and MDA-MB-231 through CRISPR-Cas9 to assess its effect on CDK4. As anticipated, USP11 deficiency remarkably reduced CDK4 protein levels (Figs. 2F, S3A). Simultaneously, the ubiquitination of CDK4 increased apparently in USP11 KO cells (Fig. S3B). Treatment with the USP11 inhibitor MTX significantly enhanced CDK4 ubiquitination (Figs. 2G, S3C). Additionally, protein half-life assays using cycloheximide (CHX) showed that exogenous USP11 markedly delayed CDK4 degradation (Fig. S3D), whereas USP11 knockout accelerated this process (Fig. S3E). Furthermore, inhibition of USP11 by MTX resulted in a decreased half-life of CDK4 (Fig. 2H). Given that USP11 has been reported to regulate p21 and E2F1 [31, 32], we examined whether USP11 affects these substrates in breast cancer cells. However, USP11 knockout did not significantly alter the ubiquitination or total protein levels of endogenous p21 or E2F1 (Fig. S3F–I). Taken together, these data establish that USP11 stabilizes CDK4 levels through its DUB activity.

### The lactylation of USP11 is required for its deubiquitination of CDK4

**Fig. 3:**
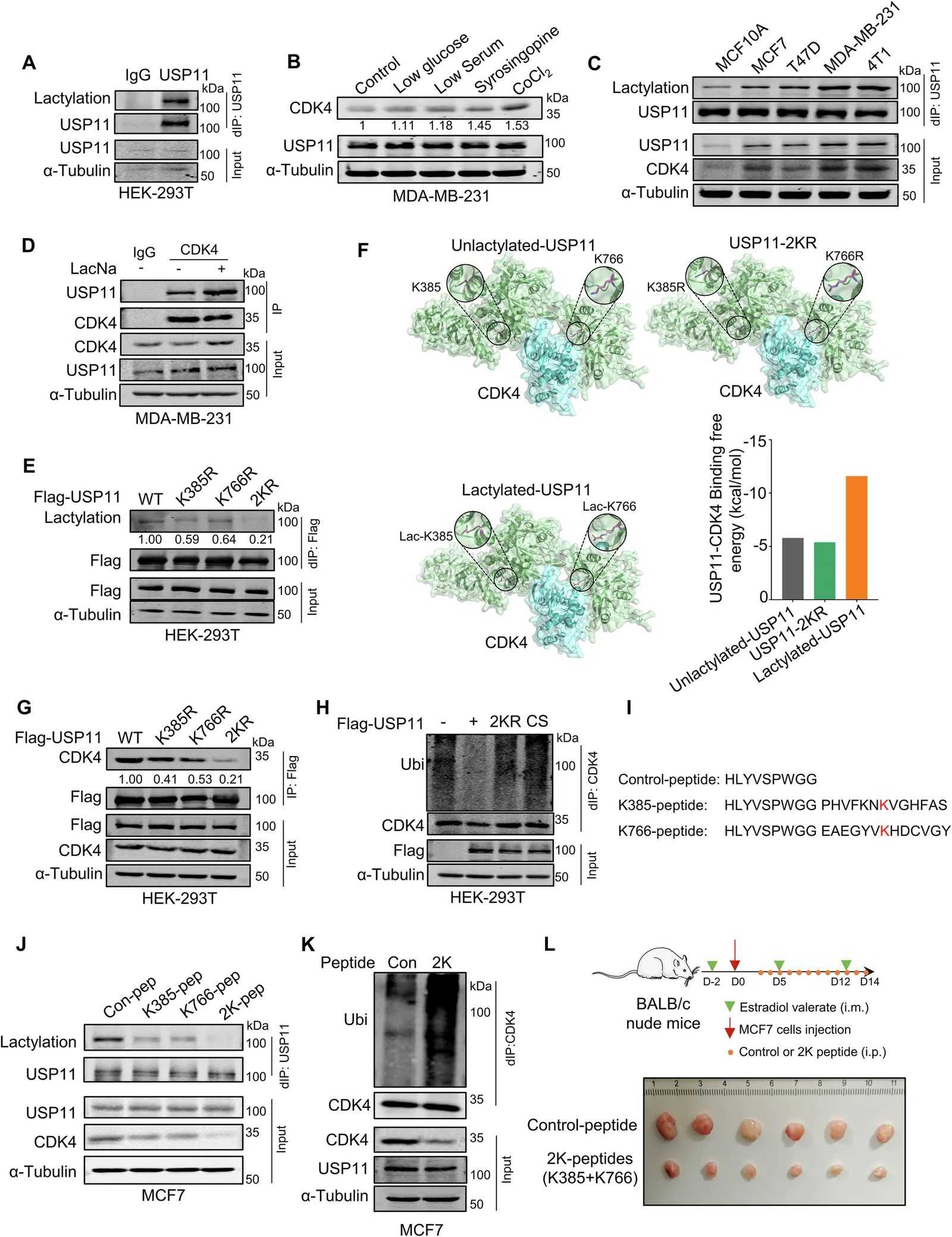
Lactylation of USP11 is essential for its ability to deubiquitinate CDK4. **A** The lactylation of USP11 was confirmed through dIP. **B** The effects of various tumor microenvironmental conditions on CDK4 protein level were investigated. Specifically, low glucose conditions were simulated using MEM with 5 mM glucose for 24 h. High lactate conditions were established by treating with 10 μM syrosingopine for 12 h. Low serum conditions were achieved by culturing cells in medium containing 1% serum for 24 h. Hypoxia was induced using CoCl_2_ at a concentration of 50 μM for 12 h. **C** The lactylation levels of USP11 in MCF10A, MCF7, T47D, MDA-MB-231, and 4T1 were detected by dIP. **D** The interaction between USP11 and CDK4 was detected by immunoprecipitation following treatment with sodium lactate (NaLac). **E** Identification of the specific lactylation sites on USP11. Flag- USP11 WT and its mutants K385R, K766R, and 2KR were individually transfected into HEK-293T cells, and the lactylation status of USP11 and these mutants was subsequently detected using dIP. **F** The binding free energy between CDK4 and USP11 in its unlactylated form, 2KR mutant, and lactylated form were measured. **G** The interactions between USP11 WT, USP11 KR/2KR mutant, and CDK4 were detected by co-IP. **H** The impact of USP11 lactylation on its ability to deubiquitinate CDK4 was investigated in HEK-293T cells transfected with Flag-tagged USP11 WT and its mutants K385/766 R and C318S. The ubiquitination of CDK4 was detected by dIP assays. **I** Sequences of the interfering peptides targeting USP11 lactylation at K385 and K766. The amino acid sequences are shown, with the lysine residue (**K**) indicated in red. A control peptide (Ctrl) was used as a negative control. **J** Representative dIP showing the effect of USP11 lactylation-targeting interfering peptides (K385-pep, K766-pep, or their combination 2K-pep) on USP11 lactylation levels in MCF7 cells. **K** dIP analysis of CDK4 ubiquitination in MCF7 cells treated with the indicated interfering peptides. **L** Photographs of MCF7 xenograft tumors harvested from mice treated with either control peptide or the combination of K385 and K766 interfering peptides (*n* = 6).

Next, we sought to identify upstream regulators of USP11, focusing on post-translational mechanisms since its overexpression did not markedly increase endogenous CDK4. To test this hypothesis, we transfected HEK-293T cells with Flag-USP11 and performed dIP, which revealed that USP11 undergoes lactylation (Fig. 3A). We next investigated the effect of tumor microenvironmental stressors on CDK4. Treatment with CoCl₂ (to mimic hypoxia) or with syrosingopine (to inhibit lactate transport) elevated endogenous CDK4 levels without affecting USP11 abundance (Fig. 3B). These conditions elevate intracellular lactate. Consistent with this, dIP showed that both sodium lactate and CoCl_2_ remarkably enhanced USP11 lactylation (Figs. S4A, S4B). Collectively, these data demonstrate that USP11 is a substrate for lactylation.

To investigate the pathophysiological relevance of USP11 lactylation, we examined its basal levels across a series of mammary epithelial cell lines. USP11 lactylation was readily detectable in both normal and malignant breast cell lines; however, its levels exhibited a marked positive correlation with the degree of cellular malignancy. Specifically, USP11 lactylation levels were lowest in normal MCF10A cells, moderate in luminal breast cancer cell lines MCF7 and T47D, and highest in aggressive TNBC lines MDA-MB-231 and 4T1, showing a positive correlation with CDK4 protein expression. Notably, this gradient of USP11 lactylation across different cell lines aligned well with the endogenous protein expression levels of CDK4 (Fig. 3C). Then, we detected the influence of USP11 lactylation on its interaction with CDK4. Sodium lactate treatment markedly strengthened the interaction between CDK4 and USP11 (Fig. 3D). Subsequently, we tried to identify the specific lactylation sites on USP11. Based on DeepKla prediction, we identified lysine residues 385 and 766 near the USP11 binding pocket (Fig. S4C). Mutating these sites reduced USP11 lactylation, with the K385/766 R (2KR) double mutant showing minimum lactylation (Fig. 3E). Molecular docking studies of CDK4 with unlactylated, lactylated, and non-lactylatable USP11-2KR revealed that lactylated USP11 had the lowest binding free energy (Fig. 3F). Consistently, the 2KR mutation disrupted the USP11-CDK4 interaction (Fig. 3G) and impaired CDK4 deubiquitination (Fig. 3H).

To directly test whether USP11 lactylation is required for its tumor‑promoting function in vivo, we designed and synthesized interfering peptides targeting the K385 and K766 lactylation sites (Fig. 3I). Treatment of MCF7 and MDA‑MB‑231 cells with these peptides, either alone or in combination, markedly reduced the lactylation signal of USP11, decreased CDK4 protein abundance (Fig. 3J, S4D), and enhanced CDK4 ubiquitination (Fig. 3K). In contrast, a control peptide had no such effects. We then administered the combination of K385 and K766 interfering peptides to mice bearing MCF7 xenografts. After two weeks of intraperitoneal injection, tumor growth was significantly suppressed in the peptide‑treated group compared with the control peptide group, as evidenced by reduction in both tumor volume and weight (Figs. 3L, S4E and S4F).

These results demonstrate that USP11 lactylation is essential for its deubiquitinating activity toward CDK4.

### USP11 knockout inhibits the activation of CDK4-RB1-E2F1 signaling pathway

**Fig. 4:**
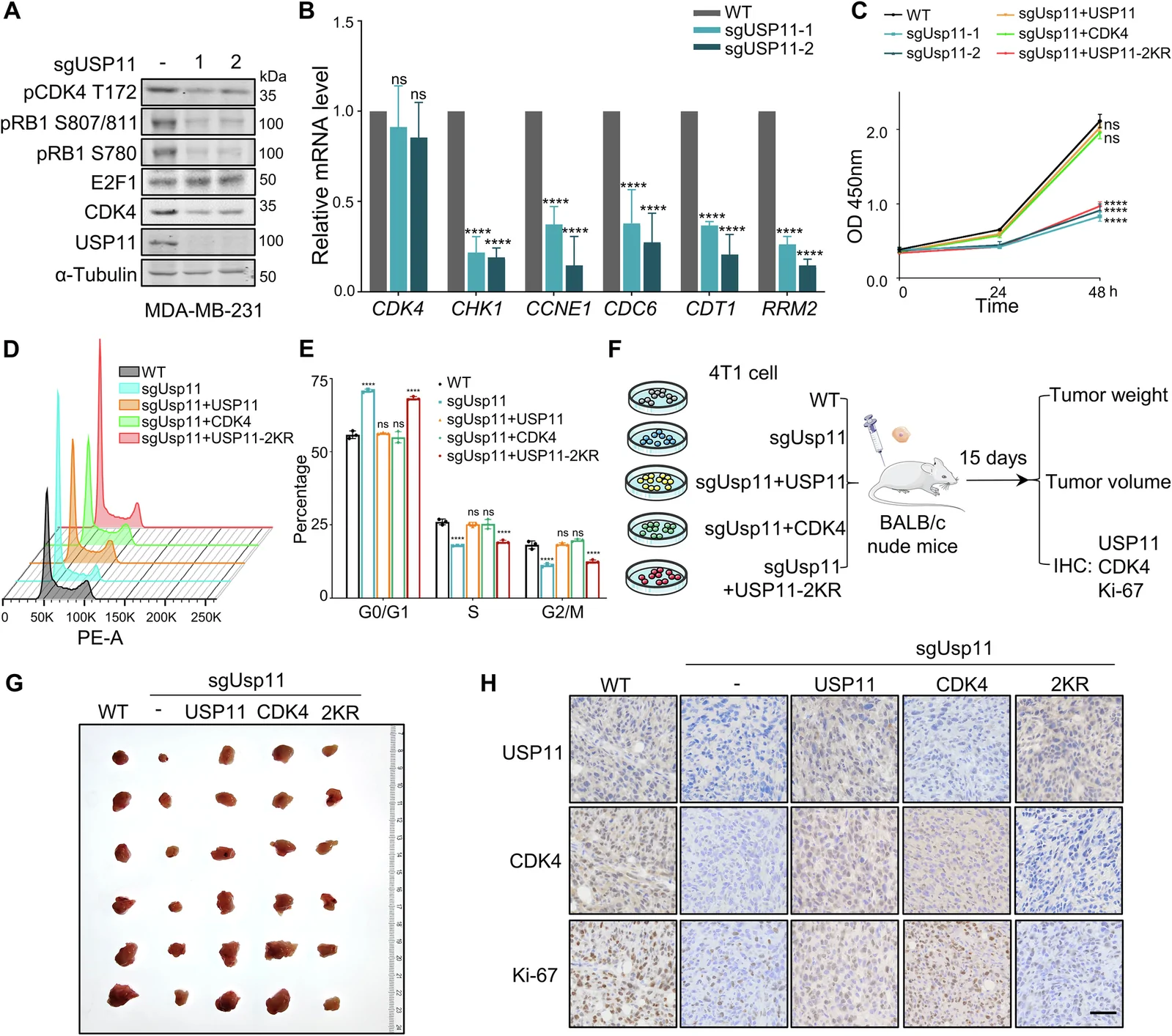
USP11 knockout suppresses the activation of the CDK4-RB1-E2F1 signaling pathway. **A** The effects of USP11 depletion on the phosphorylation status of CDK4 at T172, RB1 at S780, and RB1 at S807/811 in MDA-MB-231 cells were detected using immunoblotting with specific antibodies. **B** The influence of USP11 KO on the mRNA expression levels of *CDK4* and its downstream target genes, including *CHK1*, *Cyclin E*, *CDC6*, *CDT1*, and *RRM2*, in MDA-MB-231 cells was determined using qRT-PCR. The results were normalized to *β-actin* levels for accuracy. The data are presented as mean ± SEM with three replicates (*n* = 3). Statistical analysis was accomplished using two-way analysis of variance (ANOVA). ns, indicates not significant (*P* > 0.05). * denotes *P* < 0.05. **, *P* < 0.01. ***, *P* < 0.001. ****, *P* < 0.0001. **C** The effect of Usp11 KO on cell proliferation was assessed in 4T1 cells. Additionally, the influence of rescuing USP11 WT, USP11-2KR, and CDK4 on cell proliferation was measured. Statistical analysis was performed using one-way ANOVA. ns, not significant (*P* > 0.05). *, *P* < 0.05. **, *P* < 0.01. ***, *P* < 0.001. ****, *P* < 0.0001. **D** The impact of Usp11 KO on cell cycle was determined in 4T1 cells by flow cytometry. **E** The distribution of 4T1 cells across various phases of the cell cycle was determined using flow cytometry. The data are presented as mean ± SEM with three biological replicates (*n* = 3). Statistical analysis was determined using Student’s *t* test. ns indicates not significant (*P* > 0.05). *, *P* < 0.05. **, *P* < 0.01. ***, *P* < 0.001. ****, *P* < 0.0001. **F**, **G** WT 4T1 cells, Usp11 KO 4T1 cells, and Usp11 KO 4T1 cells rescued with USP11, CDK4, or USP11-2KR were separately injected into BALB/c nude mice. After 15 days, tumor weight and tumor volume were measured. Additionally, the protein expression levels of USP11, CDK4, and Ki-67 were detected by IHC. Photographs of the tumors from each group (*n* = 6) are presented in (**G**). **H** The protein expression levels of USP11, CDK4 and Ki-67 in tumors were detected by IHC.

To detect the influence of USP11 on CDK4 activation and the G1 phase progression, we assessed the phosphorylation levels of CDK4 (T172) and RB1 (S780, S807/811) in WT cells and USP11 KO cells. USP11 knockout in both MCF7 and MDA-MB-231 cell lines markedly decreased phosphorylation of CDK4 at T172 and RB1 at S780/S807/811 compared to WT cells (Figs. 4A, S5A), suggesting that USP11 is necessary for CDK4 activation.

The CDK4/6-RB1-E2F1 pathway is essential for G1/S phase transition, ultimately driving the expression of E2F1 target genes required for S phase entry. To explore the impact of USP11 ablation on this pathway, we examined the expression of key E2F1 target genes. Consistent with impaired CDK4/RB1 phosphorylation, these genes were significantly downregulated following USP11 KO (Fig. 4B). Notably, quantitative RT PCR analysis revealed that USP11 knockout did not significantly alter CDK4 mRNA levels, indicating that USP11 regulates CDK4 expression at the post-translational level rather than through transcriptional control. Collectively, these results demonstrate that USP11 loss suppresses CDK4 activation and impedes G1 progression.

We next assessed whether USP11 knockout impairs cell proliferation, given its role in CDK4 activation. CCK8 assay showed markedly reduced proliferation in USP11 KO MCF7 and MDA-MB-231 cells compared to wild-type controls (Figs. S5B, S5C). To confirm the specificity of this effect, we reintroduced USP11, CDK4, or USP11-2KR mutant into USP11 KO 4T1 cells (Figs. S5D, S5E). Overexpression of USP11 or CDK4 rescued the proliferation defect induced by USP11 KO, whereas USP11-2KR did not (Fig. 4C). Furthermore, flow cytometry analysis following PI staining revealed a significant G1 phase arrest in USP11 KO cells (Fig. 4D, E). Together, these findings demonstrate that USP11 is essential for G1 progression and proliferation.

Next, to evaluate the effect of USP11 on tumorigenesis in vivo, we established a syngeneic mouse model (Fig. 4F). Tumors were harvested and measured 15 days after subcutaneous injection (Fig. 4G). USP11 knockout significantly suppressed tumor growth, as evidenced by reduced tumor volume, weight, and growth kinetics. This suppression was rescued by re-expression of either wild-type USP11 or CDK4, but not by the USP11-2KR mutant (Fig. S5F–H). Consistent with the growth data, IHC analysis showed that Ki-67 expression was restored in tumors from USP11 KO cells re-expressing USP11 or CDK4 (Fig. 4H).

To further investigate the functional significance of the USP11‑CDK4 axis in ER+ luminal breast cancer, we reconstituted USP11 knockout (KO) MCF7 cells with wild‑type USP11, wild‑type CDK4, or the lactylation‑deficient mutant USP11‑2KR (Fig. S6A). CCK8 proliferation assays showed that USP11 KO significantly reduced the proliferation rate. Reconstitution with either USP11 or CDK4 effectively restored proliferation to levels comparable to those of control cells, whereas reconstitution with USP11‑2KR failed to rescue this defect (Fig. S6B). Cell cycle analysis further revealed that USP11 KO induced a pronounced G1 arrest, which was similarly rescued by USP11 or CDK4, but not by USP11‑2KR (Fig. S6C, D).

To validate these findings under physiologically relevant conditions, we subcutaneously injected the above cell lines into 6‑week‑old female BALB/c nude mice pretreated with estradiol valerate to mimic the hormonal environment of ER+ breast cancer. Two weeks post‑injection, tumor measurements showed that the USP11 KO group formed significantly smaller and lighter tumors compared with the control group. Reconstitution with USP11‑WT or CDK4‑WT restored tumor growth to levels comparable to those of control tumors, whereas reconstitution with USP11‑2KR had no significant effect (Fig. S6E–G).

Having established that USP11 promotes proliferation and tumor growth in ER+ luminal breast cancer cells by stabilizing CDK4, we next investigated whether USP11 might regulate CDK4 through additional mechanisms beyond direct deubiquitination. To address this, we examined the potential effects of USP11 knockout on the subcellular localization of CDK4, the interaction between CDK4 and Cyclin D1, and the interaction between CDK4 and SKP2. Our results showed that USP11 knockout did not alter the distribution pattern of CDK4 between the nucleus and cytoplasm, as determined by immunofluorescence assays, indicating that USP11 does not affect CDK4 subcellular localization (Figs. S6H, S6I). Furthermore, co‑IP experiments revealed that USP11 depletion did not affect the binding between CDK4 and Cyclin D1. Similarly, USP11 knockout did not alter the interaction between CDK4 and SKP2, a known E3 ligase for CDK4 (Fig. S6J). Taken together, these findings suggest that, in breast cancer cells, USP11 primarily regulates CDK4 protein stability through direct deubiquitination, rather than by altering its subcellular localization or its interactions with Cyclin D1 or SKP2. Collectively, these results confirm that USP11 is essential for tumor growth in vivo in a manner dependent on its deubiquitinase activity and CDK4.

### USP11 and CDK4 are highly expressed in breast cancer and are correlated with an unfavorable prognosis

**Fig. 5:**
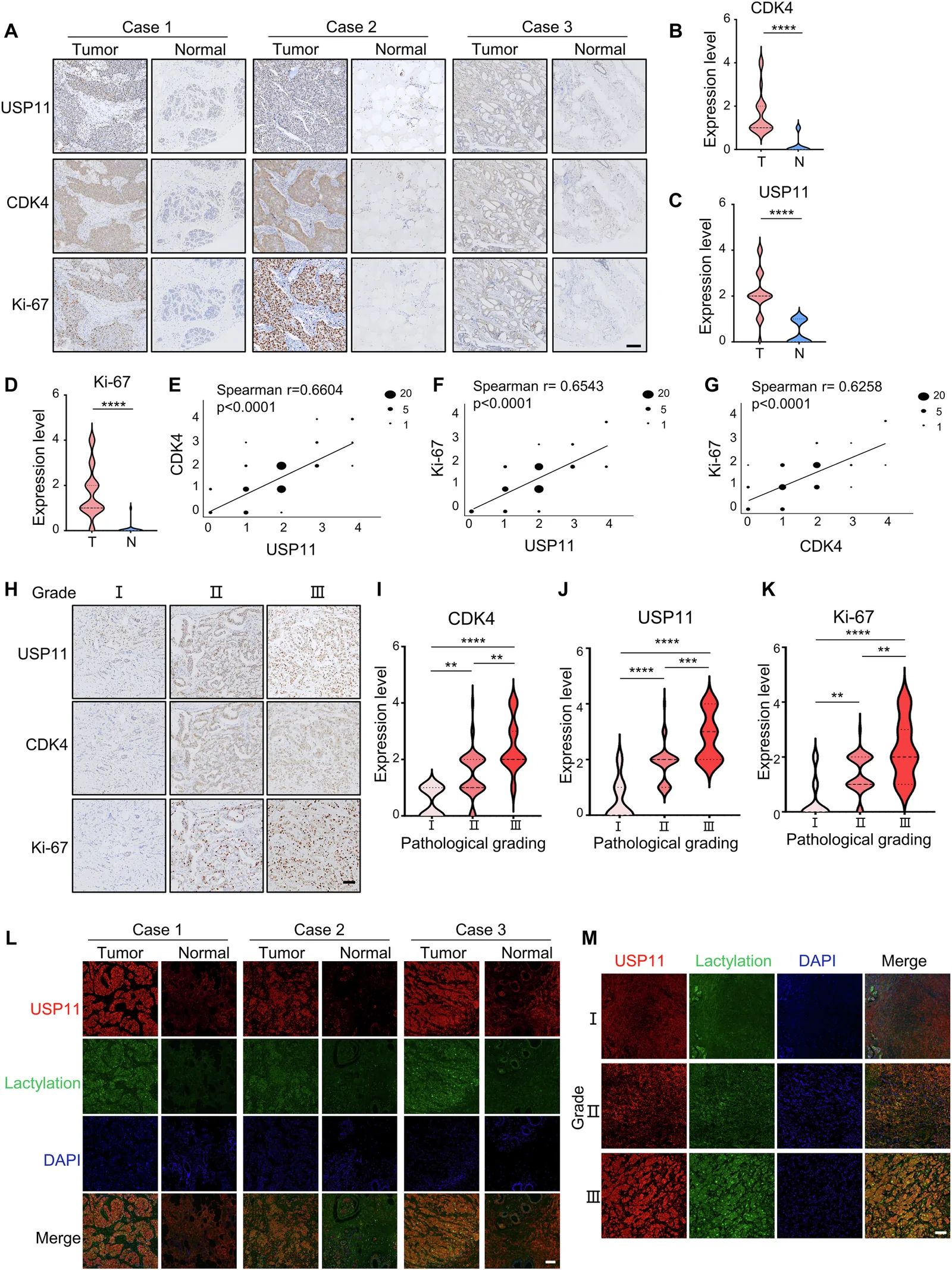
USP11 and CDK4 are both highly expressed in breast cancer and are associated with a poor prognosis. **A** The IHC staining was performed to evaluate the expression levels of USP11, CDK4, and Ki-67 in both tumor and corresponding normal tissues from patients. Scale bar, 100 μm. The expression levels of CDK4 (**B**), USP11 (**C**), and Ki-67 (**D**), quantified by histochemical scoring, were assessed in both tumor and normal tissues from a cohort of 20 patients (*n* = 20). T: Tumor tissue. N: Normal tissue. The correlations between USP11 and CDK4 expression levels (**E**), USP11 and Ki-67 expression levels (**F**), and between CDK4 and Ki-67 expression levels (**G**) were determined using IHC staining data. **H** IHC staining was performed to evaluate the expression levels of USP11, CDK4, and Ki-67 in tumor tissues from breast cancer patients, spanning different pathological grades. Scale bar, 100 μm. The expression levels of CDK4 (**I**), USP11 (**J**), and Ki-67 (**K**), as indicated by histochemical scores, were evaluated across pathological grades I (*n* = 8), II (*n* = 50), and III (*n* = 11). **L** Representative multiplex immunofluorescence images showing the colocalization of USP11 (red) and lactylation (green) in adjacent normal and breast tumor tissues. Nuclei were counterstained with DAPI (blue). Merged images indicate colocalization (yellow). Scale bar, 100 μm. **M** Representative multiplex immunofluorescence images of USP11 (red) and lactylation (green) colocalization in breast tumor tissues across pathological grades I (*n* = 8), II (*n* = 50), and III (*n* = 11). Nuclei were counterstained with DAPI (blue). Merged images indicate colocalization (yellow). Scale bar, 100 μm.

To evaluate the clinical relevance of USP11 and CDK4 in breast cancer, we analyzed TCGA BRCA database and found that elevated levels of USP11 and CDK4 expression are significantly associated with poor patient prognosis (Figs. S7A, S7B). Consistent with this, immunohistochemical analysis of clinical samples revealed that USP11, CDK4, and the proliferation marker Ki-67 were markedly upregulated in BRCA tumor tissues compared to adjacent normal tissues (Fig. 5A–D). Furthermore, we assessed the correlation between protein expression and pathological grading. Protein expression levels of USP11, CDK4, and Ki-67 showed significant positive correlations with each other (Fig. 5E–G) and increased with advancing pathological grade (Fig. 5H–K).

To further evaluate the clinical relevance of USP11 lactylation, we performed multiplex immunofluorescence analysis of USP11 and lactylation on a tissue microarray containing 69 breast cancer samples. Compared with adjacent normal tissues, tumor tissues exhibited significantly stronger colocalization of USP11 with lactylation signals (Figs. 5L, S7C). Further analysis demonstrated that USP11 lactylation levels were positively correlated with tumor pathological grade, with higher‑grade tumors exhibiting stronger USP11 lactylation signals (Figs. 5M, S7D). Moreover, the intensity of colocalization was positively correlated with CDK4 protein expression levels (Fig. S7E). To directly detect endogenous USP11 lactylation at the in situ level, we established a Duolink PLA using an anti‑USP11 antibody in combination with an anti‑lactyl‑lysine antibody. PLA analysis of tumor tissue sections revealed distinct USP11 lactylation signals within the tumor tissues, directly confirming the presence of endogenous USP11 lactylation in breast tumors. Importantly, no PLA signals were detected in USP11‑knockout tumors, whereas reconstitution with wild‑type USP11 restored the PLA signals. In contrast, reconstitution with the lactylation‑deficient USP11‑2KR mutant failed to generate detectable PLA signals (Fig. S7F), further validating the specificity of the assay and the critical role of K385/K766 lactylation. Quantitative analysis revealed that the majority of PLA signals were localized in the cytoplasm, indicating that USP11 lactylation predominantly occurs in the cytoplasmic compartment (Fig. S7G). These results support the clinical significance of the USP11-CDK4 axis in breast cancer progression and prognosis.

### The synergistic administration of USP11 inhibitor and CDK4 inhibitor has a more potent antitumor effect

**Fig. 6:**
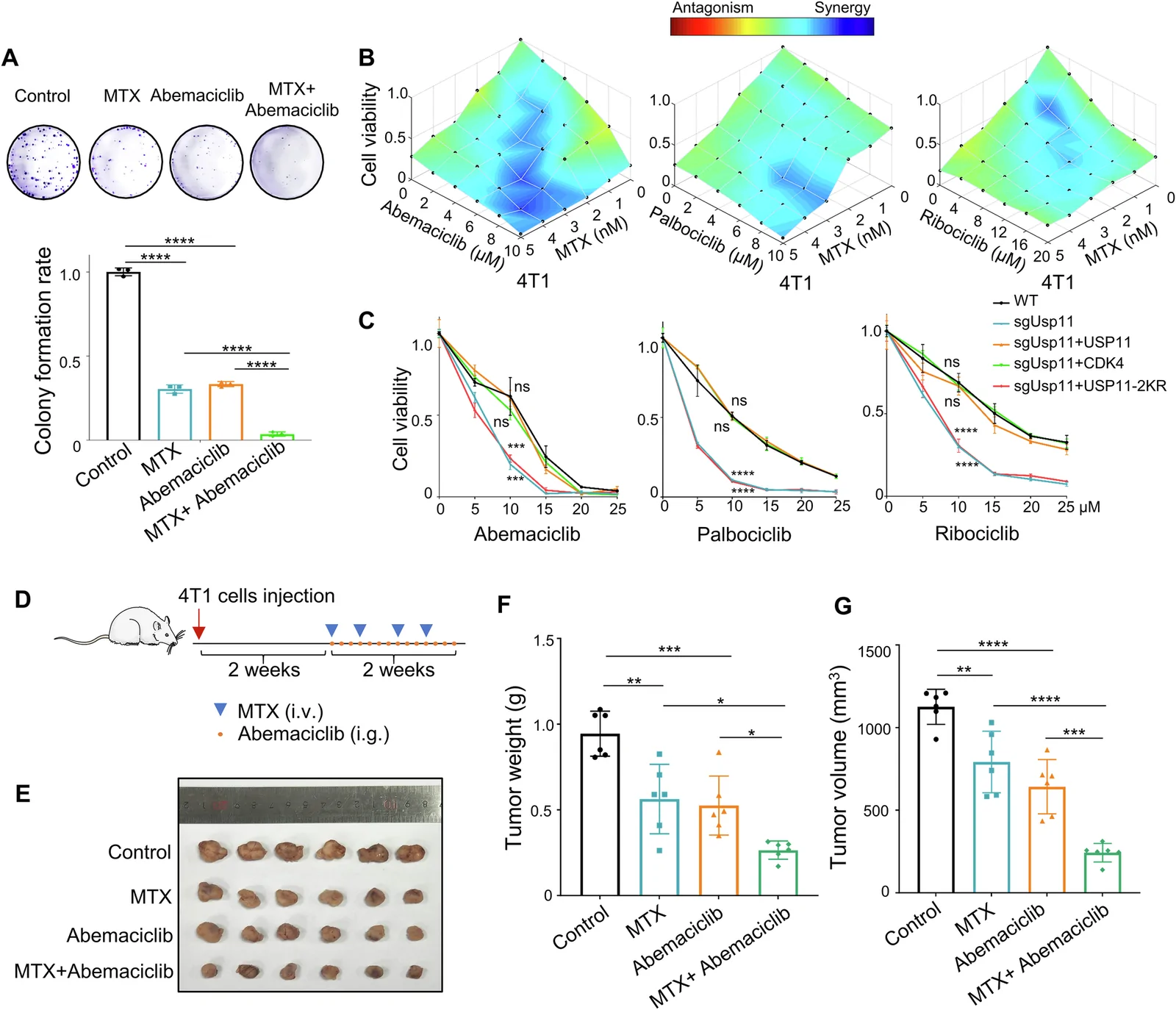
The combination of USP11 inhibitor and CDK4 inhibitor exhibits a stronger antitumor effect. **A** Colony formation assays were conducted to test the cytotoxic effects of MTX and Abemaciclib, both individually and in combination, on MDA-MB-231 cells. MTX was applied at concentration of 10 nM, while Abemaciclib was used at concentration of 5 μM for 24 h. The relative colony formation rates were presented. Statistical analysis was performed using one-way ANOVA. ns, not significant (*P* > 0.05). *, *P* < 0.05. **, *P* < 0.01. ***, *P* < 0.001. ****, *P* < 0.0001. **B** The in vitro synergetic antitumor effects of MTX in combination with Abemaciclib, Palbociclib, or Ribociclib were evaluated across a range of concentrations using the CCK8 assay in 4T1 cells. The graphical representation of the data was generated by Combenefit software. **C** The sensitivities of USP11 KO 4T1 cells to Abemaciclib, Palbociclib, and Ribociclib were assessed. Additionally, the effects of rescuing USP11, USP11-2KR mutant, and CDK4 in 4T1 cells on these sensitivities were measured. Statistical analysis was performed using one-way ANOVA. ns, not significant (*P* > 0.05). *, *P* < 0.05. **, *P* < 0.01. ***, *P* < 0.001. ****, *P* < 0.0001. **D**, **E** Balb/c nude mice were subcutaneously injected with 4T1 cells. After 2 weeks, the mice were randomly divided into four groups and treated with either saline, MTX, Abemaciclib, or a combination of both inhibitors. The treatment regimen lasted for an additional two weeks, after which the mice were euthanized (**D**). Photographs of the tumors from each group (*n* = 6) were presented in (**E**). Tumor weight (**F**) and tumor volume (**G**) of each group from (**E**) were measured. Statistical analysis was performed using two-way ANOVA. ns not significant (*P* > 0.05). *, *P* < 0.05. **, *P* < 0.01. ***, *P* < 0.001. ****, *P* < 0.0001.

Our research demonstrated that USP11 deficiency reduces both the quantity and activity of CDK4. Given that CDK4 inhibitors are clinically effective in ER-positive breast cancer but exhibit limited efficacy in TNBC, we hypothesized that targeting USP11 could sensitize RB-proficient TNBC to these agents. We therefore tested whether the combination of USP11 inhibitor MTX and CDK4 inhibitor Abemaciclib achieves superior therapeutic outcomes in RB-proficient TNBC. Colony formation assay in MDA-MB-231 cells revealed a concentration-dependent reduction in clonogenicity, with the combination treatment showing significantly greater efficacy than either agent alone (Fig. 6A). This synergetic antitumor effect was further confirmed for Abemaciclib, Palbociclib, and Ribociclib across a range of concentration in vitro (Fig. 6B, S8A–C). Consistent with this, CCK8 assays demonstrated that USP11 knockout enhanced the sensitivity of MDA-MB-231 and 4T1 cells to CDK4 inhibitors (Fig. S8D). This acquired sensitivity was rescued by re-expression of wild-type USP11 or CDK4, but not by the USP11-2KR mutant (Fig. 6C). The combination of MTX and Abemaciclib nearly abolished RB1 S780 phosphorylation, whereas monotherapy with either agent alone showed only a partial inhibitory effect (Fig. S8E).

To evaluate the synergetic antitumor efficacy of MTX and CDK4 inhibitor in vivo, we generated a BRCA mouse model through the subcutaneous injection of 4T1 cells. After two weeks, these mice were randomly assigned to four groups and treated with either saline, MTX, Abemaciclib, or a combination of both inhibitors. The treatment continued for an additional two weeks, after which the mice were sacrificed (Fig. 6D). Necropsy revealed that treatment with MTX or Abemaciclib alone led to a reduction in tumor volume and weight. Notably, the combination group therapy resulted in the greatest reduction, with tumors exhibiting the smallest size and lowest weight compared to the control or single-agent groups (Fig. 6E–G). Collectively, these findings demonstrate that the combination of a USP11 inhibitor with a CDK4 inhibitor has a superior antitumor effect in RB-proficient TNBC models both in vitro and in vivo.

## Discussion

**Fig. 7:**
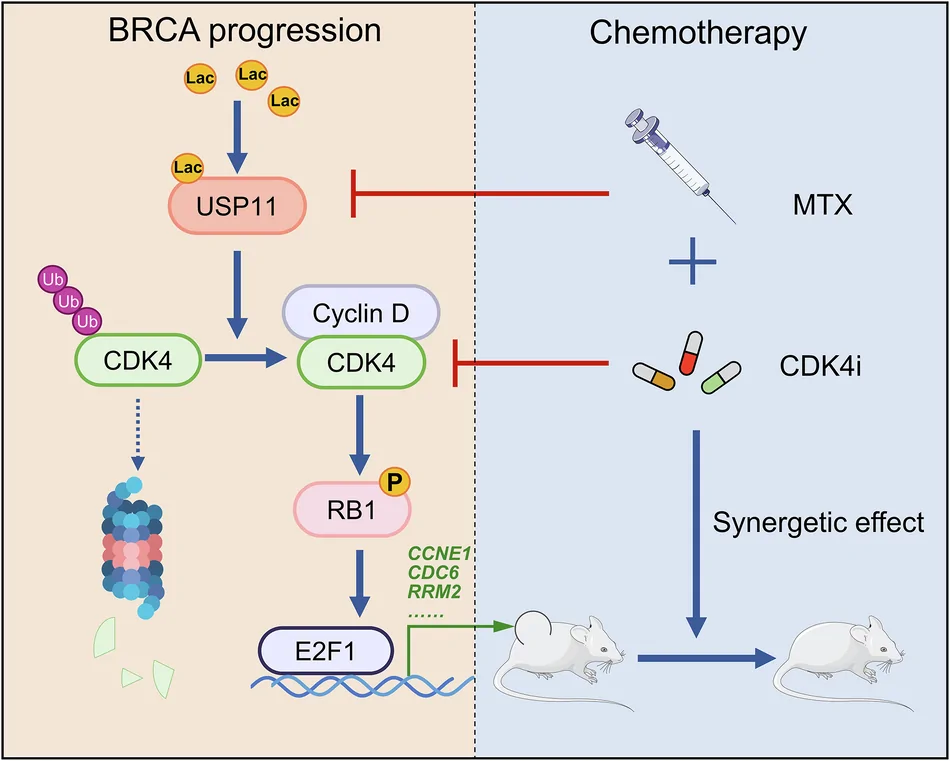
A working model for deubiquitinase USP11 promotes breast cancer tumor progression by stabilizing CDK4. Lactate, generated by the Warburg effect, promotes lactylation of USP11. This modification enhances the deubiquitination and stability of CDK4. Consequently, USP11 deficiency significantly reduces CDK4 abundance, thereby inhibiting the cell cycle progression and proliferation. The combination of CDK4 and USP11 inhibitors exhibits synergistic efficacy in breast cancer treatment, providing new therapeutic strategies for combating cancer. Lac Lactate, Ubi Ubiquitin, P Phosphorylation.

Tumor cells are characterized by dysregulated cell cycle control and unlimited proliferation potential [6]. As a central regulator of the G1/S phase transition and a core component of the CDK4/6–RB1–E2F1 signaling axis, CDK4 plays a pivotal role in the pathogenesis of multiple cancer types [3–5]. In many cancers, CDK4 is frequently overexpressed, and its levels often correlate with disease severity [12–15]. Notably, recent study suggests that elevated CDK4 expression contributes to resistance against CDK4 inhibitors [19–21]. Therefore, targeting the protein levels of CDK4 could provide novel therapeutic strategies for cancer treatment. This study demonstrates that USP11 promotes breast cancer progression by stabilizing CDK4 and activating the CDK4-RB1-E2F1 pathway. Targeting this axis with a combination of USP11 and CDK4 inhibitors resulted in a more potent antitumor effect (Fig. 7).

Owing to the periodic expression of cyclins, their ubiquitination-mediated degradation has been extensively characterized. For example, the ubiquitination of cyclin D has been widely reported [36–39]. In contrast, the ubiquitination of CDKs has not been as extensively investigated, primarily due to their relatively constant expression levels throughout the cell cycle. Although ubiquitination-mediated degradation of CDK4, CDK6, and CDK2 has been reported [22, 40, 41], the mechanisms that stabilize these proteins remain poorly understood, particularly with regard to deubiquitination processes. Here, our research demonstrated that the ubiquitination of CDK4 is specifically removed by USP11, thereby maintaining its protein level. Conversely, USP11 deficiency led to a significant reduction of CDK4, which in turn significantly impaired cell proliferation.

The present study demonstrates that USP11 promotes breast cancer progression by stabilizing CDK4. This finding contrasts with reports in which USP11 was shown to stabilize p21 to suppress tumorigenesis in lung and colon cancer cells [31], or to stabilize E2F1 to drive proliferation in hepatocellular carcinoma [32]. We propose that these discrepancies reflect the context-dependent functionality of USP11. Potential explanations for the observed differences include variations in cell type and tumor-driving pathways, as well as distinctions in the tumor microenvironment and post-translational modifications. Notably, in our experimental system, USP11 knockout did not alter the ubiquitination or protein levels of p21 or E2F1 (Fig. S3F–I), indicating that CDK4, rather than these factors, is the predominant USP11 target in breast cancer cells under the studied conditions. It is important to note that Deng et al. primarily investigated lung cancer and colon cancer cells, Qiao et al. focused on hepatocellular carcinoma, while the present study centers on breast cancer. Tumors originating from different tissues exhibit distinct core oncogenic drivers and metabolic profiles. Notably, our study reveals for the first time that USP11 itself is regulated by lactylation. In the Warburg effect-enriched breast cancer microenvironment, elevated lactate levels induce lactylation of USP11 at residues K385 and K766, which enhances its binding to CDK4 and strengthens its deubiquitinating activity. This metabolic modification may redirect USP11 function toward CDK4 stabilization, providing a mechanistic explanation for the observed context-dependent substrate selectivity.

USP11 is localized on the X chromosome, and previous studies have revealed significant gender-specific differences in its expression [42, 43], potentially due to X chromosome inactivation or estrogen-induced regulation, which may underlie certain female-predominant diseases [34, 35]. In breast cancer, this sex-specific expression pattern is particularly noteworthy, as the estrogen signaling pathway plays a central role in mammary tumorigenesis and progression. It is therefore reasonable to speculate that USP11 may function downstream of estrogen signaling to promote breast cancer progression, and that estrogen deprivation therapies might also affect USP11 expression or activity. In our research, exogenous overexpression of USP11 did not further increase CDK4 levels, suggesting that endogenous USP11 is already sufficient to maintain CDK4 stability. This could be a protective evolutionary mechanism for CDK4 to ensure cell proliferation. Accordingly, USP11 depletion markedly reduced CDK4 levels and suppressed breast cancer cell proliferation, indicating that the USP11-CDK4 interaction is subject to precise regulation. Our findings also partially answered the regulatory mechanisms of USP11 by PTMs. Like many deubiquitinases, USP11 may be modulated by modifications that allow rapid adaptation to cellular signals [44]. In recent studies, lactylation has been widely detected within cells [45, 46]. Here, for the first time, we demonstrated that deubiquitinase can be lactylated and investigate how this modification affects its interaction with substrates. Lactylation may neutralize positive charges on lysine residues, thereby altering protein-protein interactions. While CDK4 overexpression in tumors has traditionally been attributed to gene amplification, our findings reveal an additional mechanism involving the Warburg effect, linking metabolic reprogramming to CDK4 stability. While targeted degradation strategies for CDK4 are currently unavailable, our study unveils critical upstream regulatory mechanisms and proposes a novel combination therapy aimed at jointly inhibiting USP11 and CDK4, offering a promising direction for therapeutic intervention.

Beyond its traditional role as a topoisomerase II inhibitor, MTX has emerged as a potent inhibitor of USP11. This drug is currently used to treat acute leukemia, breast cancer, lymphoma, and other tumors [47, 48]. Combining CDK4/6 inhibitors with topoisomerase II inhibitors presents a therapeutic challenge, as CDK4/6 inhibition-induced G1 arrest can protect cells from the cytotoxic effects of topoisomerase II inhibitors, which is most effective in S/G2/M phase. However, we found that MTX, owing to its USP11 inhibitory activity, enables a synergistic strategy. Co-administering MTX with a CDK4 inhibitor delivers a “second hit” to the CDK4-RB1-E2F1 pathway, thereby enhancing antitumor efficacy.

Our study revealed that USP11 expression is elevated in breast cancer tissues and further increases with disease progression. Previous studies indicate that cells with higher USP11 levels exhibit greater sensitivity to MTX [47]. Given that CDK4 kinase activity is essential for breast cancer development [17], CDK4 inhibitors have been incorporated into clinical treatment regimens, including for certain TNBC cases [49]. And combining CDK4 inhibitors with PD-1 antibodies can improve therapeutic outcomes [50, 51]. Notably, resistance to CDK4 inhibitors has been linked to elevated CDK4 expression, which can be further induced by the inhibitors themselves [19–21]. Our results indicate that pharmacological inhibition of USP11 with MTX reduces CDK4 protein levels, suggesting a potential strategy to overcome such resistance. This approach may also allow for dose reduction of CDK4/6 inhibitors, thereby potentially minimizing associated toxicity. We note that approximately 30% of TNBC patients harbor RB loss or mutations, which may render them insensitive to CDK4 inhibitors. Therefore, the proposed combination therapy of a USP11 inhibitor and a CDK4 inhibitor is likely most applicable to breast cancer patients with intact RB function, including ER+ luminal breast cancer and RB-proficient TNBC subtypes. Molecular stratification based on RB status will be an important consideration for precision medicine in clinical translation. The recent development of selective CDK4 inhibitors such as Atirmociclib, designed to reduce off-target toxicity [52], further supports the feasibility of this combined targeting strategy.

Our complementary experiments demonstrated that USP11 primarily regulates CDK4 protein stability through direct deubiquitination, rather than by altering its subcellular localization or its interactions with Cyclin D1 or SKP2. Nevertheless, we acknowledge that USP11 is a multifunctional deubiquitinase. Therefore, we cannot completely exclude the possibility that other unidentified substrates or parallel pathways may partially contribute to the observed cell cycle phenotypes. Future studies using proteomic approaches to comprehensively map USP11 substrates in breast cancer cells will help to further delineate the precise contribution of the USP11‑CDK4 axis relative to other potential effectors.

In conclusion, our study demonstrates that USP11 promotes breast cancer progression by deubiquitinating and stabilizing CDK4 at residues K88, K106, and K142, leading to activation of the CDK4/6-RB1-E2F signaling pathway. The combination therapy of USP11 inhibitor MTX with CDK4 inhibitors demonstrates superior antitumor activity, highlighting its potential as an effective treatment strategy.

## Materials and methods

### Animal models

All animal studies were authorized and performed following the animal guidelines by the IACUC of the Center for Experimental Animal Research (China) and Peking University Laboratory Animal Center (IACUC No. LSC-ZhengXF-1). Four-week-old female BALB/c nude mice (Jiangsu Gempharmatech Laboratory Animal Technology) were injected subcutaneously in the flank with 4T1 cells (1 × 10^6^ cells per mouse). Two weeks after injection, MTX was administered intravenously (5 mg/kg), while Abemaciclib was given orally via intragastric gavage (90 mg/kg). The tumor volume was calculated using the formula: volume = length × width^2^/2. Female BALB/c nude mice, 6 weeks of age, were obtained from Jiangsu GemPharmatech Co., Ltd.

To establish the estrogen‑responsive MCF7 xenograft model, mice received an intramuscular injection of estradiol valerate (2 mg/kg) two days prior to tumor cell inoculation, followed by repeated injections once every seven days thereafter to maintain a physiological estrogen level. A total of 1 × 10⁷ MCF7 cells were subcutaneously injected into the right flank of each mouse. For peptide treatment, mice were randomly assigned to control or treatment groups. Starting from day 3 post‑inoculation, the peptides were administered by daily intraperitoneal injection at a dose of 5 mg/kg. All mice were euthanized two weeks after tumor cell inoculation, and tumors were excised, weighed, photographed, and processed for further histological or biochemical analyses.

### Statistical analyses

All statistical results are performed as the mean ± SEM of three or more independent biological replicates. Statistical analysis was accomplished by Student’s *t* test, one-way ANOVA or two-way ANOVA using GraphPad Prism 10.1 software (ns, *P* > 0.05. *, *P* < 0.05. **, *P* < 0.01. ***, *P* < 0.001. ****, *P* < 0.0001).

Additional materials and methods including plasmids, cell lines, Reagents, antibodies, Statistical analyses, and all other assays are described in Supplementary Information, SI Materials and Methods.

## Supplementary information

Supplementary Materials & Methods, Figures S1-S8 & legends

Original Western Blot

## Data Availability

The mass spectrometry proteomics data have been deposited to the ProteomeXchange Consortium via the PRIDE partner repository with the dataset identifier PXD064603 and 10.6019/PXD064603.

## References

[1] Matsushime H, Ewen ME, Strom DK, Kato JY, Hanks SK, Roussel MF, et al. Identification and properties of an atypical catalytic subunit (p34PSK-J3/cdk4) for mammalian D type G1 cyclins. Cell. 1992;71:323–34. 10.1016/0092-8674(92)90360-o 1423597

[2] Kato J, Matsushime H, Hiebert SW, Ewen ME, Sherr CJ. Direct binding of cyclin D to the retinoblastoma gene product (pRb) and pRb phosphorylation by the cyclin D-dependent kinase CDK4. Genes Dev. 1993;7:331–42. 10.1101/gad.7.3.331 8449399

[3] Meyerson M, Harlow E. Identification of G1 kinase activity for cdk6, a novel cyclin D partner. Mol Cell Biol. 1994;14:2077–86. 10.1128/mcb.14.3.2077-2086.1994 PMC358568 8114739

[4] Matsushime H, Quelle DE, Shurtleff SA, Shibuya M, Sherr CJ, Kato JY. D-type cyclin-dependent kinase activity in mammalian cells. Mol Cell Biol. 1994;14:2066–76. 10.1128/mcb.14.3.2066-2076.1994 PMC358567 8114738

[5] Kato JY, Matsuoka M, Strom DK, Sherr CJ. Regulation of cyclin D-dependent kinase 4 (cdk4) by cdk4-activating kinase. Mol Cell Biol. 1994;14:2713–21. 10.1128/mcb.14.4.2713-2721.1994 PMC358637 8139570

[6] Hanahan D, Weinberg RA. Hallmarks of cancer: the next generation. Cell. 2011;144:646–74. 10.1016/j.cell.2011.02.013 21376230

[7] Fry DW, Harvey PJ, Keller PR, Elliott WL, Meade M, Trachet E, et al. Specific inhibition of cyclin-dependent kinase 4/6 by PD 0332991 and associated antitumor activity in human tumor xenografts. Mol Cancer Ther. 2004;3:1427–38. 15542782

[8] Prat A, Solovieff N, Andre F, O’Shaughnessy J, Cameron DA, Janni W, et al. Intrinsic subtype and overall survival of patients with advanced HR+/HER2- breast cancer treated with ribociclib and ET: correlative analysis of MONALEESA-2, -3, -7. Clin Cancer Res. 2024;30:793–802. 10.1158/1078-0432.CCR-23-0561 PMC10870119 37939142

[9] Lu YS, Im SA, Colleoni M, Franke F, Bardia A, Cardoso F, et al. Updated overall survival of ribociclib plus endocrine therapy versus endocrine therapy alone in pre- and perimenopausal patients with HR+/HER2- advanced breast cancer in MONALEESA-7: a phase III randomized clinical trial. Clin Cancer Res. 2022;28:851–9. 10.1158/1078-0432.CCR-21-3032 PMC9377723 34965945

[10] Goetz MP, Toi M, Huober J, Sohn J, Tredan O, Park IH, et al. Abemaciclib plus a nonsteroidal aromatase inhibitor as initial therapy for HR+, HER2- advanced breast cancer: final overall survival results of MONARCH 3. Ann Oncol. 2024;35:718–27. 10.1016/j.annonc.2024.04.013 38729566

[11] Burger C, Wick M, Muller R. Lineage-specific regulation of cell cycle gene expression in differentiating myeloid cells. J Cell Sci. 1994;107:2047–54. 10.1242/jcs.107.7.2047 7983166

[12] An HX, Beckmann MW, Reifenberger G, Bender HG, Niederacher D. Gene amplification and overexpression of CDK4 in sporadic breast carcinomas is associated with high tumor cell proliferation. Am J Pathol. 1999;154:113–8. 10.1016/S0002-9440(10)65257-1 PMC1868599 9916925

[13] Miliani de Marval PL, Macias E, Conti CJ, Rodriguez-Puebla ML. Enhanced malignant tumorigenesis in Cdk4 transgenic mice. Oncogene. 2004;23:1863–73. 10.1038/sj.onc.1207309 PMC2859671 14647432

[14] Barretina J, Taylor BS, Banerji S, Ramos AH, Lagos-Quintana M, Decarolis PL, et al. Subtype-specific genomic alterations define new targets for soft-tissue sarcoma therapy. Nat Genet. 2010;42:715–21. 10.1038/ng.619 PMC2911503 20601955

[15] Cancer Genome Atlas Research N, Brat DJ, Verhaak RG, Aldape KD, Yung WK, Salama SR, et al. Cancer Genome Atlas Research N Comprehensive, integrative genomic analysis of diffuse lower-grade gliomas. N Engl J Med. 2015;372:2481–98. 10.1056/NEJMoa1402121 PMC4530011 26061751

[16] Reddy HK, Mettus RV, Rane SG, Grana X, Litvin J, Reddy EP. Cyclin-dependent kinase 4 expression is essential for neu-induced breast tumorigenesis. Cancer Res. 2005;65:10174–8. 10.1158/0008-5472.CAN-05-2639 16288002

[17] Yu Q, Sicinska E, Geng Y, Ahnstrom M, Zagozdzon A, Kong Y, et al. Requirement for CDK4 kinase function in breast cancer. Cancer Cell. 2006;9:23–32. 10.1016/j.ccr.2005.12.012 16413469

[18] Hu MG, Deshpande A, Enos M, Mao D, Hinds EA, Hu GF, et al. A requirement for cyclin-dependent kinase 6 in thymocyte development and tumorigenesis. Cancer Res. 2009;69:810–8. 10.1158/0008-5472.CAN-08-2473 PMC2636510 19155308

[19] Olanich ME, Sun W, Hewitt SM, Abdullaev Z, Pack SD, Barr FG. CDK4 amplification reduces sensitivity to CDK4/6 inhibition in fusion-positive rhabdomyosarcoma. Clin Cancer Res. 2015;21:4947–59. 10.1158/1078-0432.CCR-14-2955 PMC4583342 25810375

[20] Cen L, Carlson BL, Schroeder MA, Ostrem JL, Kitange GJ, Mladek AC, et al. p16-Cdk4-Rb axis controls sensitivity to a cyclin-dependent kinase inhibitor PD0332991 in glioblastoma xenograft cells. Neuro Oncol. 2012;14:870–81. 10.1093/neuonc/nos114 PMC3379801 22711607

[21] Cai Z, Wang J, Li Y, Shi Q, Jin L, Li S, et al. Overexpressed Cyclin D1 and CDK4 proteins are responsible for the resistance to CDK4/6 inhibitor in breast cancer that can be reversed by PI3K/mTOR inhibitors. Sci China Life Sci. 2023;66:94–109. 10.1007/s11427-021-2140-8 35982377

[22] Wang L, Zhang R, You X, Zhang H, Wei S, Cheng T, et al. The steady-state level of CDK4 protein is regulated by antagonistic actions between PAQR4 and SKP2 and involved in tumorigenesis. J Mol Cell Biol. 2017;9:409–21. 10.1093/jmcb/mjx028 28992327

[23] Yilmaz D, Furst A, Meaburn K, Lezaja A, Wen Y, Altmeyer M, et al. Activation of homologous recombination in G1 preserves centromeric integrity. Nature. 2021;600:748–53. 10.1038/s41586-021-04200-z 34853474

[24] Ting X, Xia L, Yang J, He L, Si W, Shang Y, et al. USP11 acts as a histone deubiquitinase functioning in chromatin reorganization during DNA repair. Nucleic Acids Res. 2019;47:9721–40. 10.1093/nar/gkz726 PMC6765148 31504778

[25] Ideguchi H, Ueda A, Tanaka M, Yang J, Tsuji T, Ohno S, et al. Structural and functional characterization of the USP11 deubiquitinating enzyme, which interacts with the RanGTP-associated protein RanBPM. Biochem J. 2002;367:87–95. 10.1042/BJ20011851 PMC1222860 12084015

[26] Sun W, Tan X, Shi Y, Xu G, Mao R, Gu X, et al. USP11 negatively regulates TNFalpha-induced NF-kappaB activation by targeting on IkappaBalpha. Cell Signal. 2010;22:386–94. 10.1016/j.cellsig.2009.10.008 PMC2794974 19874889

[27] Al-Salihi MA, Herhaus L, Macartney T, Sapkota GP. USP11 augments TGFbeta signalling by deubiquitylating ALK5. Open Biol. 2012;2:120063. 10.1098/rsob.120063 PMC3390794 22773947

[28] Garcia DA, Baek C, Estrada MV, Tysl T, Bennett EJ, Yang J, et al. USP11 Enhances TGFbeta-Induced Epithelial-Mesenchymal Plasticity and Human Breast Cancer Metastasis. Mol Cancer Res. 2018;16:1172–84. 10.1158/1541-7786.MCR-17-0723 PMC6030438 29724812

[29] Lee EW, Seong D, Seo J, Jeong M, Lee HK, Song J. USP11-dependent selective cIAP2 deubiquitylation and stabilization determine sensitivity to Smac mimetics. Cell Death Differ. 2015;22:1463–76. 10.1038/cdd.2014.234 PMC4532773 25613375

[30] Zhou Z, Luo A, Shrivastava I, He M, Huang Y, Bahar I, et al. Regulation of XIAP turnover reveals a role for USP11 in promotion of tumorigenesis. EBioMedicine. 2017;15:48–61. 10.1016/j.ebiom.2016.12.014 PMC5233825 28040451

[31] Deng T, Yan G, Song X, Xie L, Zhou Y, Li J, et al. Deubiquitylation and stabilization of p21 by USP11 is critical for cell-cycle progression and DNA damage responses. Proc Natl Acad Sci USA. 2018;115:4678–83. 10.1073/pnas.1714938115 PMC5939064 29666278

[32] Qiao L, Zhang Q, Sun Z, Liu Q, Wu Z, Hu W, et al. The E2F1/USP11 positive feedback loop promotes hepatocellular carcinoma metastasis and inhibits autophagy by activating ERK/mTOR pathway. Cancer Lett. 2021;514:63–78. 10.1016/j.canlet.2021.05.015 34044068

[33] Park MK, Yao Y, Xia W, Setijono SR, Kim JH, Vila IK, et al. PTEN self-regulates through USP11 via the PI3K-FOXO pathway to stabilize tumor suppression. Nat Commun. 2019;10:636. 10.1038/s41467-019-08481-x PMC6367354 30733438

[34] Dwane L, O’Connor AE, Das S, Moran B, Mulrane L, Pinto-Fernandez A, et al. A functional genomic screen identifies the deubiquitinase USP11 as a novel transcriptional regulator of ERalpha in breast cancer. Cancer Res. 2020;80:5076–88. 10.1158/0008-5472.CAN-20-0214 33004351

[35] Yan Y, Wang X, Chaput D, Shin MK, Koh Y, Gan L, et al. X-linked ubiquitin-specific peptidase 11 increases tauopathy vulnerability in women. Cell. 2022;185:3913–30.e3919. 10.1016/j.cell.2022.09.002 PMC9588697 36198316

[36] Qie S, Diehl JA. Cyclin D degradation by E3 ligases in cancer progression and treatment. Semin Cancer Biol. 2020;67:159–70. 10.1016/j.semcancer.2020.01.012 PMC7390705 32006569

[37] Chaikovsky AC, Li C, Jeng EE, Loebell S, Lee MC, Murray CW, et al. The AMBRA1 E3 ligase adaptor regulates the stability of cyclin D. Nature. 2021;592:794–8. 10.1038/s41586-021-03474-7 PMC8246597 33854239

[38] Maiani E, Milletti G, Nazio F, Holdgaard SG, Bartkova J, Rizza S, et al. AMBRA1 regulates cyclin D to guard S-phase entry and genomic integrity. Nature. 2021;592:799–803. 10.1038/s41586-021-03422-5 PMC8864551 33854232

[39] Simoneschi D, Rona G, Zhou N, Jeong YT, Jiang S, Milletti G, et al. CRL4(AMBRA1) is a master regulator of D-type cyclins. Nature. 2021;592:789–93. 10.1038/s41586-021-03445-y PMC8875297 33854235

[40] Bellail AC, Olson JJ, Hao C. SUMO1 modification stabilizes CDK6 protein and drives the cell cycle and glioblastoma progression. Nat Commun. 2014;5:4234. 10.1038/ncomms5234 PMC4090607 24953629

[41] Ying M, Shao X, Jing H, Liu Y, Qi X, Cao J, et al. Ubiquitin-dependent degradation of CDK2 drives the therapeutic differentiation of AML by targeting PRDX2. Blood. 2018;131:2698–711. 10.1182/blood-2017-10-813139 29720484

[42] Tukiainen T, Villani AC, Yen A, Rivas MA, Marshall JL, Satija R, et al. Landscape of X chromosome inactivation across human tissues. Nature. 2017;550:244–8. 10.1038/nature24265 PMC5685192 29022598

[43] Li X, Cui XL, Wang JQ, Wang YK, Li YF, Wang LY, et al. Generation and Application of Mouse-Rat Allodiploid Embryonic Stem Cells. Cell. 2016;164:279–92. 10.1016/j.cell.2015.11.035 26771496

[44] Sahtoe DD, Sixma TK. Layers of DUB regulation. Trends Biochem Sci. 2015;40:456–67. 10.1016/j.tibs.2015.05.002 26073511

[45] Zhang D, Tang Z, Huang H, Zhou G, Cui C, Weng Y, et al. Metabolic regulation of gene expression by histone lactylation. Nature. 2019;574:575–80. 10.1038/s41586-019-1678-1 PMC6818755 31645732

[46] Li H, Sun L, Gao P, Hu H. Lactylation in cancer: current understanding and challenges. Cancer Cell. 2024;42:1803–7. 10.1016/j.ccell.2024.09.006 39393355

[47] Burkhart RA, Peng Y, Norris ZA, Tholey RM, Talbott VA, Liang Q, et al. Mitoxantrone targets human ubiquitin-specific peptidase 11 (USP11) and is a potent inhibitor of pancreatic cancer cell survival. Mol Cancer Res. 2013;11:901–11. 10.1158/1541-7786.MCR-12-0699 23696131

[48] Liao Y, Zhou D, Wang P, Yang M, Jiang N. Ubiquitin specific peptidase 11 as a novel therapeutic target for cancer management. Cell Death Discov. 2022;8:292. 10.1038/s41420-022-01083-5 PMC9205893 35715413

[49] Jiang YZ, Ma D, Suo C, Shi J, Xue M, Hu X, et al. Genomic and transcriptomic landscape of triple-negative breast cancers: subtypes and treatment strategies. Cancer Cell. 2019;35:428–40.e425. 10.1016/j.ccell.2019.02.001 30853353

[50] Zhang J, Bu X, Wang H, Zhu Y, Geng Y, Nihira NT, et al. Cyclin D-CDK4 kinase destabilizes PD-L1 via cullin 3-SPOP to control cancer immune surveillance. Nature. 2018;553:91–95. 10.1038/nature25015 PMC5754234 29160310

[51] Yu J, Yan J, Guo Q, Chi Z, Tang B, Zheng B, et al. Genetic aberrations in the CDK4 pathway are associated with innate resistance to PD-1 blockade in Chinese patients with non-cutaneous melanoma. Clin Cancer Res. 2019;25:6511–23. 10.1158/1078-0432.CCR-19-0475 31375512

[52] Palmer CL, Boras B, Pascual B, Li N, Li D, Garza S, et al. CDK4 selective inhibition improves preclinical anti-tumor efficacy and safety. Cancer Cell. 2025;43:464–81.e414. 10.1016/j.ccell.2025.02.006 40068598

