# Supplementary material for: Lactylation of USP11 fosters breast cancer development by stabilizing CDK4 and activating CDK4-RB1-E2F1 pathway
Source: Cell Death Dis. 2026 Aug 12;17(1):955. doi: 10.1038/s41419-026-09172-4 (PMC13748568; doi:10.1038/s41419-026-09172-4)
Supplement: Supplementary file 1 [file 41419_2026_9172_MOESM1_ESM.pdf]

Supplementary Information
SI Methods
Supplementary figures and legends 1–8
SI References
Supplementary File 1: original western blots

SI Materials and Methods

Plasmids

The cDNAs of human CDK4 and USP11 were amplified and cloned into pCDNA3.0-3Flag, pCDNA3.0-3Myc, pGEX4T1, and pET28a vectors, respectively. CDK4 was also amplified and cloned into pCMV-HA vector. The Ubiquitin gene was cloned into pEF1-HisA and pCMV-HA vectors. All CDK4 and USP11 mutants were generated through site-directed mutagenesis. The mutants of ubiquitin were kind gifts from Prof. Lingqiang Zhang. All these plasmids were verified by DNA sequencing.

Reagents and antibodies

Syrosingopine (TN2252) and proteasome inhibitor MG132 (T2154) were purchased from TargetMol. L-Sodium lactate (71718) was purchased from Sigma. MTX dihydrochloride (S2485) was purchased from Selleck. Cycloheximide (HY-12320), Abemaciclib methanesulfonate (HY-16297), Palbociclib hydrochloride (HY-50767C), and Ribociclib hydrochloride (HY-15777A) were purchased from MedChemExpress (MCE).

Flag antibody (F3165, RRID: AB_259529) and HA antibody (H3663, RRID: AB_262051) were purchased from Sigma. His antibody (D291-3, RRID: AB_10597733) and GST antibody (M071-3, RRID: AB_591784) were purchased from MBL. Mouse monoclonal Myc Antibody (EM31105, RRID: AB_3068994) was purchased from HUABIO. Mouse monoclonal α-Tubulin antibody (AC012, RRID: AB_2768341) was purchased from ABclonal. Rabbit IgG antibody (2729S, RRID: AB_1031062) was purchased from Cell Signaling Technology (CST). Rabbit polyclonal CDK4 antibody (11026-1-AP, RRID: AB_2078702), mouse monoclonal CDK4 antibody (66950-1-Ig, RRID: AB_2882273), rabbit polyclonal USP11 antibody (22340-1-AP, RRID: AB_2879079), mouse monoclonal USP11 antibody (67805-1-Ig, RRID: AB_2918568) and mouse monoclonal RB1 antibody (67521-1-Ig, RRID: AB_2882741) were purchased from Proteintech. Rabbit monoclonal β-actin was purchased from Abclonal (AC004, RRID: AB_2737399). Mouse monoclonal ubiquitin antibody (P4D1, RRID: AB_10998070) was purchased from ENZO. L-lactyl lysine (PTM-1401RM, RRID: AB_2942013) was purchased from PTM Biolab. Rabbit polyclonal Phospho-CDK4 (Thr172) Antibody (AF5767) was purchased from Beyotime. Rabbit monoclonal Phospho-Rb (Ser780) antibody (8180, RRID: AB_10950972) and rabbit monoclonal Phospho-Rb (Ser807/811) antibody (9308, RRID: AB_331472) were purchased from CST. All primary antibodies were diluted at 1:1000. IRDye 800CW goat anti-mouse (926-32210, RRID: AB_3096013) and IRDye 800CW goat anti-rabbit (926-32211, RRID: AB_621843) were purchased from LI-COR Biosciences. All secondary antibodies were diluted at 1:10000.

Cell culture and transfection

HEK293T, T47D, MCF7, MDA-MB-231, and 4T1 cells were cultured in Dulbecco's Modified Eagle Medium (DMEM, MeilunBio, MA0212) with 10% fetal bovine serum (MeilunBio). The MDA-MB-231 was a kind gift from Prof. Jinfang Zhang (Wuhan University). All those cells were cultured in a 37°C incubator with 5% CO_2. The identities of all cell lines were authenticated by short tandem repeat analysis and were tested by PCR to make sure there is no mycoplasma contamination. Cells were transfected using polyethylenimine (PEI, Polyscience) or Hieff Trans (YEASEN). For special conditions, low glucose conditions were simulated using MEM (MACKLIN, D6514) with 5 mM glucose (Gibco, A2494001) for 24 h. High lactate conditions were constructed using DMEM by treatment of 10 μM syrosingopine for 12 h. Low serum conditions were achieved by culturing cells in DMEM containing 1% serum for 24 h. Hypoxia was induced using $CoCl_2$ (MACKLIN, C805641) at a concentration of 50 μM for 12 h using DMEM.

Immunoprecipitation (IP)

Cells were transfected with plasmids if necessary. At 48 h after transfection, cells were collected with PBS and lysed using NP-40 buffer (50 mM Tris-HCl, pH 7.4, 150 mM NaCl, 1% NP-40 and 1 mM EDTA) with 1% protease inhibitor (MCE, HY-K0010). Cell supernatants were collected after centrifugation at 12000 rpm for 10 min at 4°C. If necessary, the supernatant could be denatured using 1% SDS. Subsequently, 50 μL of the supernatant was reserved as the input sample. The remaining supernatant was then incubated with antibodies for 4 h at 4°C. Next the protein G was added and incubated for an additional 4 h. Finally, the protein G was separated by centrifugation, washed three times with NP-40 buffer, and subsequently denatured using SDS loading buffer.

Western blot analysis

Protein samples were subjected to SDS-PAGE and then transferred onto nitrocellulose membrane (GE Healthcare, USA). The membrane was blocked with 5% non-fat milk, and incubated with the primary antibody, followed by the secondary antibody. The signals on the membrane were detected by the Odyssey Infrared Imaging System (LI-COR Biosciences, USA).

Immunofluorescence (IF)

Cells were seeded on coverslips in a 6-well plate. After treatment, cells were gently rinsed with PBS and subsequently fixed with 4% paraformaldehyde for 20 min. Next, the cells were permeabilized using 0.1% Triton X-100. Afterward, the cells were blocked with 3% BSA and incubated with the primary antibody, followed by the secondary antibody. Finally, the cells were visualized using a confocal microscope (Zeiss LSM-880). Quantification was performed using ImageJ software. The colocalization statistics were performed using the JACoP plugin [1].

Duolink proximity ligation assay (PLA)

Cells were seeded on coverslips in a 6-well plate. Following treatment, cells were rinsed with PBS, fixed with 4% paraformaldehyde for 20 min, and then permeabilized with 0.1%

Triton X-100. Subsequently the cells were blocked with blocking buffer and incubated with the primary antibody. Next, the cells were incubated with ligation buffer. After three washes using Wash Buffer A, polymerase and fluorescently labeled oligonucleotides were added to amplify the signals. After three additional washes with Wash Buffer B, images were captured using a confocal microscope (Zeiss LSM-880). Quantification was performed using ImageJ software.

Protein purification and *in vitro* pulldown assays

Recombinant His-tagged CDK4 protein was expressed in *E. coli* BL21 and purified using a HisTrap™ HP column (GE Healthcare). GST-tagged USP11 protein was also expressed in *E. coli* BL21 and purified by glutathione-Sepharose 4B column (GE Healthcare). The purified proteins were incubated together and immunoprecipitated using His or GST antibodies for *in vitro* pulldown assays.

***In vitro* deubiquitination assays**

Ubiquitinated CDK4 in HEK-293T cells was immunoprecipitated using a CDK4 antibody. The immunoprecipitated complex was then incubated with either recombinant GST-USP11 or GST-USP11-C318S purified from *E. coli*, respectively, in deubiquitinating buffer (60 mM HEPES, 5 mM $MgCl_2$, 4% glycerol, pH 7.6) at 30°C for 4 h.

Knockout cell line construction

USP11 KO cells were generated using CRISPR-Cas9 system. The following sgRNAs were used for CRISPR/Cas9-mediated knockout of USP11 in MCF7 and MDA-MB-231 cells:

USP11 sgRNA1: 5'-ATTGTTGATGCAGCCAGGGA-3'

USP11 sgRNA2: 5'-GCGTTGGCTGTAGAAGAGAA-3'

The following sgRNAs were used for CRISPR/Cas9-mediated knockout of Usp11 in 4T1 cells:

Usp11 sgRNA1: 5'-AACTACATGGTCAGCTGGTA-3'

Usp11 sgRNA2: 5'-TTTGGCGCCACTGTTCATCC-3'

Lentivirus infection

USP11, CDK4, and USP11-K385/766R were constructed into the pLVX-IRES-ZsGreen1 vector, and then packaged into the lentivirus with the pLP1, pLP2, and VSV-G vectors. Cells were infected using lentivirus and selected via flow cytometry.

Molecular Mechanics Poisson–Boltzmann Surface Area (MM/PBSA) calculations

The USP11 structure was modeled based on predictions from AlphaFold. Lysine residues K385 and K766 were mutated to arginine, resulting in the inactive form USP11-2KR, or were lactylated to form the modified version Lac-USP11. The crystal structure of CDK4 (PDB:2W96) was employed as the initial template. Given the absence of experimental co-crystal structures, the binding conformations of USP11-CDK4, USP11-2KR-CDK4, and Lac-USP11-CDK4 complexes were predicted using molecular docking via the GRAMM-X web server [2]. The binding free energy landscape was quantitatively characterized through

Molecular Mechanics/Poisson-Boltzmann Surface Area (MM/PBSA) calculations implemented in the MMPBSA.py module. This computational approach systematically identified key binding determinants by decomposing energy contributions from individual residues in both protein-ligand and protein-cofactor complexes, with particular emphasis on those exhibiting significant thermodynamic contributions [3].

Protein half-life assay

Cells were treated with cycloheximide (CHX, 50 μg/mL) to block protein synthesis. Cell lysates were collected at specified intervals. Target proteins were detected by Western blot using specific antibodies and semi-quantified using α-Tubulin as loading control.

RT-PCR and quantitative real-time PCR analyses

Total RNA was extracted from cells using TRIzol (Magen) and then reverse transcribed into cDNA (TIANGEN). Subsequently, the cDNA templates were amplified using SYBR Green Master Mix (YEASEN) according to the specific primers. Primer sequences were shown below:

ACTB (Forward): 5'- CATGTACGTTGCTATCCAGGC-3',
ACTB (Reverse): 5'- CTCCTTAATGTCACGCACGAT-3';
CDK4 (Forward): 5'- ATGGCTACCTCTCGATATGAGC -3',
CDK4 (Reverse): 5'- CATTGGGGACTCTCACACTCT -3';
CHK1 (Forward): 5'- ATATGAAGCGTGCCGTAGACT -3',
CHK1 (Reverse): 5'- TGCCTATGTCTGGCTCTATTCTG -3';
CCNE1 (Forward): 5'- AAGGAGCGGGACACCATGA -3',
CCNE1 (Reverse): 5'- ACGGTCACGTTTGCCTTCC -3';
CDC6 (Forward): 5'- CCAGGCACAGGCTACAATCAG-3',
CDC6 (Reverse): 5'- AACAGGTTACGGTTTGGACATT-3';
CDT1 (Forward): 5'- CGGTGGACGAGGTTTCCAG-3',
CDT1 (Reverse): 5'- CTGCCGGGGTGGATTTCTT-3';
RRM2 (Forward): 5'- CACGGAGCCGAAAACTAAAGC-3',
RRM2 (Reverse): 5'- TCTGCCTTCTTATACATCTGCCA-3';

Drug resistance and cell proliferation assays

Cells were plated (2000 cells/well) in 96-well plates with complete medium. The test drugs compounds were added after the cells had attached for 12 h, followed by incubation (37°C, 5% CO_2) for additional 24 h. Subsequently, CCK-8 solution (MeilunBio) was added to each well and incubated for 1 h. Finally, the absorbance at 450 nm was measured using a SpectraMax i3x plate reader (Molecular Devices). The synergetic effects were calculated by Combenefit [4].

Synergy was quantified using the Highest Single Agent (HSA) model. For each combination of drug A at concentration a and drug B at concentration b, the reference effect R(a,b) was defined as the greater effect (i.e., lower cell viability) of the two single agents at the same concentrations:

$R\_HSA(a,b) = \min(E\_A(a), E\_B(b))$

where E represents the measured fractional cell viability (relative to untreated

control).

The HSA synergy score S(a,b) was then calculated as the difference between the observed combination effect and the HSA reference:

S(a,b) = R_HSA(a,b)–E_obs(a,b)

Positive S values indicate synergy (greater-than-expected cell killing), and negative values indicate antagonism.

Cell cycle assay

Cells were plated (1× 10^5 cells/well) in 6-well plates with complete medium. Then cells were trypsinized and washed with PBS. Next, the cells were resuspended in ice-cold 70% ethanol, which was added dropwise while vortexing to ensure even distribution. And the cells were then fixed at 4°C overnight. Afterwards, the cells were treated with RNase A (100 μg/mL) and PI (50 μg/mL) and analyzed by flow cytometry (BD FACSVerse). The proportion of cells in each phase were calculated and analyzed using FlowJo.

Animal models

All animal studies were authorized and performed following the animal guidelines by the IACUC of the Center for Experimental Animal Research (China) and Peking University Laboratory Animal Center (IACUC No. LSC-ZhengXF-1). Four-week-old female BALB/c nude mice (Jiangsu Gempharmatech Laboratory Animal Technology) were injected subcutaneously in the flank with 4T1 cells (1× 10^6 cells per mouse). Two weeks after injection, MTX was administered intravenously (5 mg/kg), while Abemaciclib was given orally via intragastric gavage (90 mg/kg). The tumor volume was calculated using the formula: volume = length × $\text{width}^2/2$.

Bioinformatics analyses of overall survival and USP11/CDK4 expression

The overall survival rates were analyzed and Kaplan-Meier curves were plotted by GEPIA2 using BRCA data from TCGA/GTEx database [5].

Immunohistochemistry (IHC) and histopathological analyses

A total of 90 human breast cancer tissue specimens, commercially sourced from Shanghai Biochip, China, were utilized for this study. Immunohistochemistry staining was completed by Servicebio and the images were captured using VS200 (Olympus). Immunohistochemical staining results were independently evaluated by pathologists at Peking University Health Science Center, who were blinded to the study details, using a standardized histochemical scoring system. USP11 and CDK4 expression levels were graded on a 5-point scale: 0 (absent), 1 (weak), 2 (moderate), 3 (strong), and 4 (very strong).

Statistical analyses

All statistical results are performed as the mean ± SEM of three or more independent biological replicates. Statistical analysis was accomplished by Student’s *t* test, one-way ANOVA or two-way ANOVA using GraphPad Prism 10.1 software (ns, P > 0.05. *, P < 0.05. **, P < 0.01. ***, P < 0.001. ****, P < 0.0001).

SI Figures

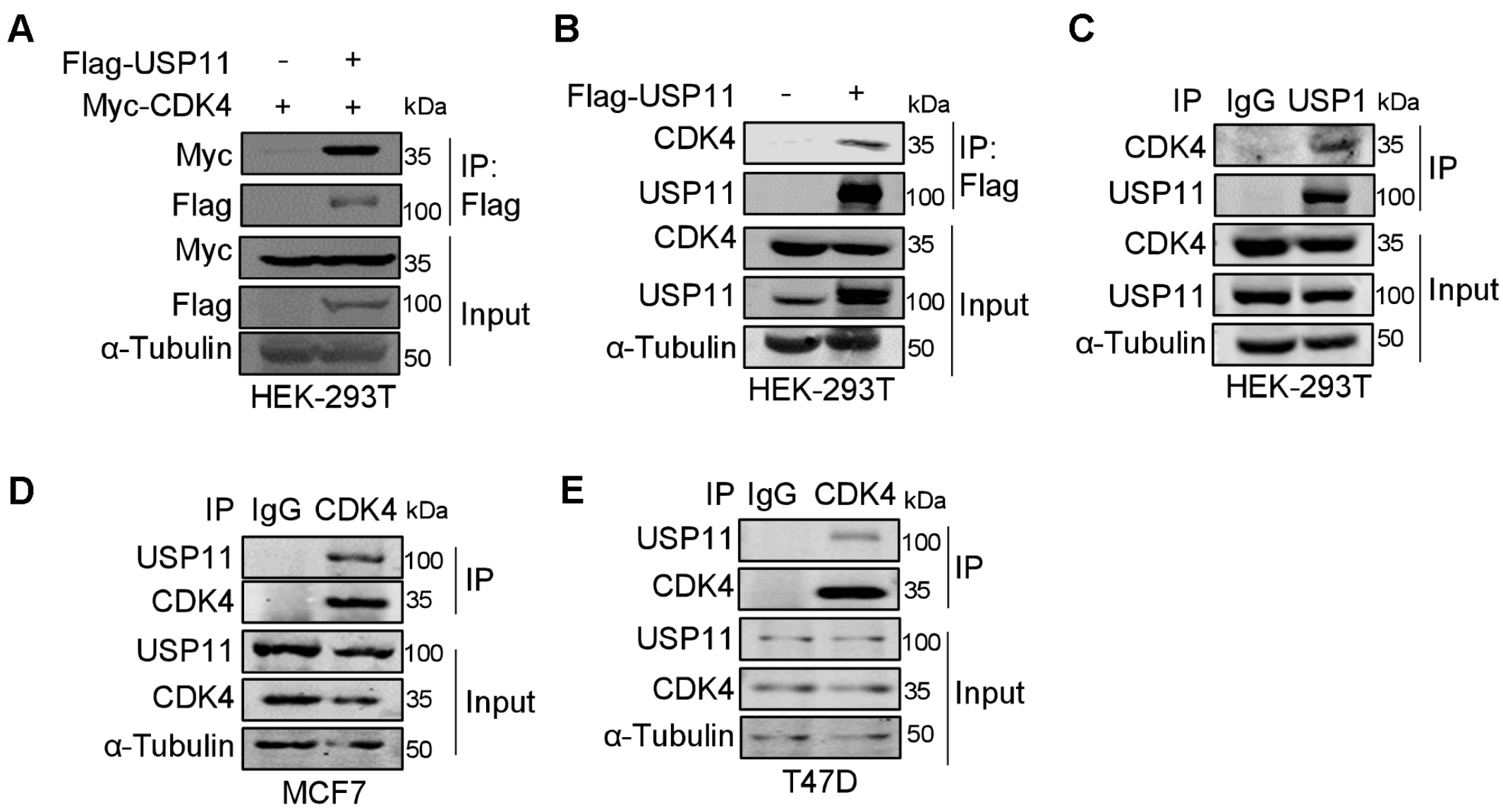

Figure S1. USP11 interacts with CDK4.

(A) HEK-293T cells transfected with Flag-USP11 and Myc-CDK4 were subjected into co-IP assay after 48 h, the proteins were enriched by anti-Flag antibody. Myc-CDK4 was co-immunoprecipitated with Flag-USP11. (B) HEK-293T cells transfected with Flag-USP11 were subjected into co-IP assay after 48 h, the proteins were enriched by anti-Flag antibody. CDK4 was co-immunoprecipitated with Flag-USP11. (C-E) The *in vivo* endogenous CDK4-USP11 interaction was detected by co-IP assays in HEK-293T cells (C), MCF7 cells (D), and T47D cells (E).

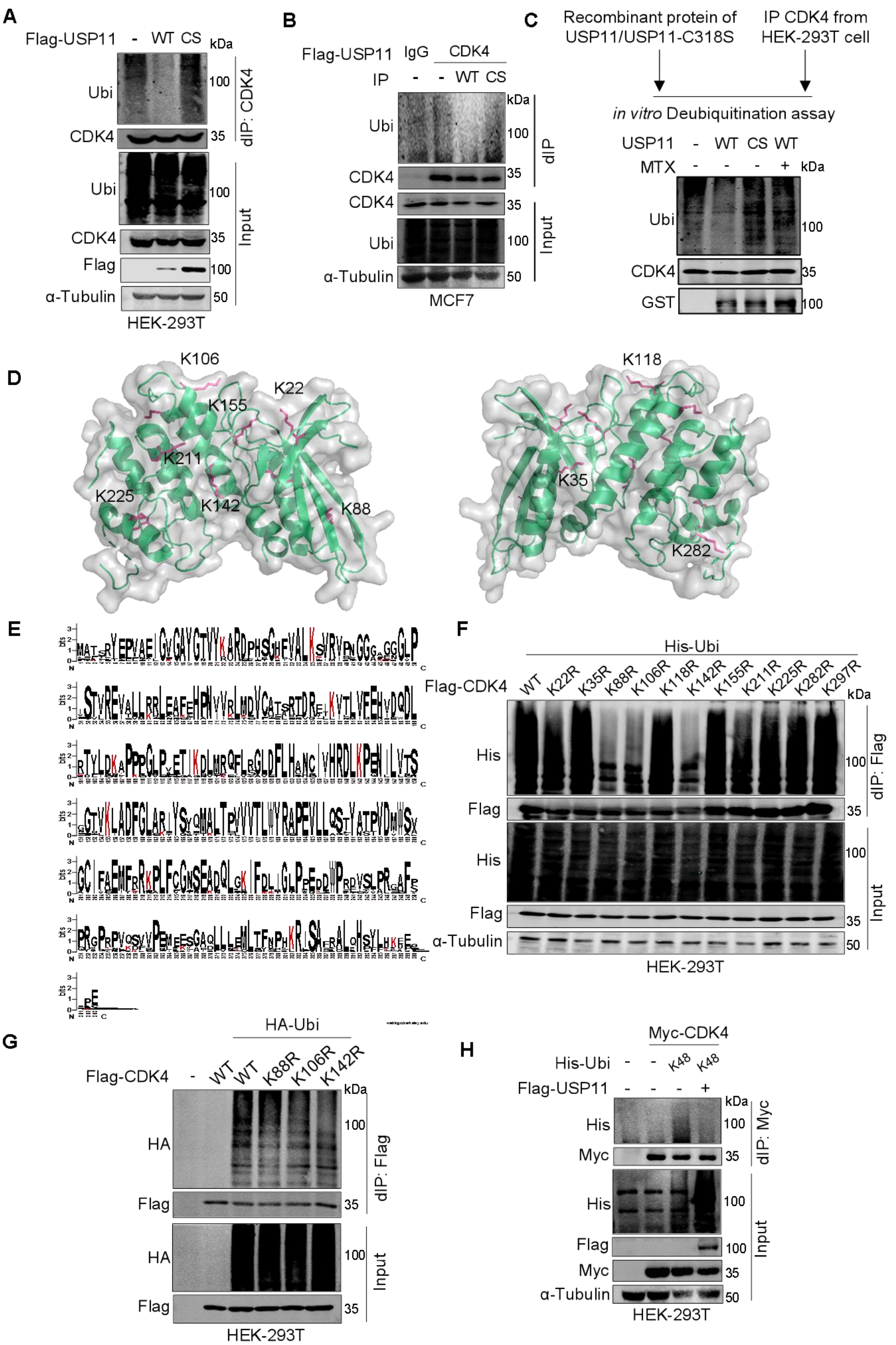

Figure S2. USP11 deubiquitinates CDK4.

(A) The effect of USP11 on endogenous CDK4 ubiquitination was assessed by denature immunoprecipitation assays in HEK-293T cells transfected with Flag-USP11 and Flag-USP11-C318S. (B) The impact of deubiquitinase activity of USP11 on CDK4

ubiquitination was investigated by denature immunoprecipitation assay in MCF7 cells transfected with Flag-USP11-WT or Flag-USP11-C318S. (C) The deubiquitination of CDK4 by USP11 was confirmed through an *in vitro* deubiquitination assay following as described in Methods. The CDK4 protein was immunoprecipitated from HEK-293T cells using specific CDK4 antibody. The USP11-WT and USP11-C318S proteins were purified from *E. coli*. Mitoxantrone (MTX) was used as an inhibitor of USP11 at a concentration of 10 nM. (D) CDK4 has 11 lysine residues, most of which are all situated on its surface. CDK4 structure was obtained from Protein Data Bank (PDB ID: 2W9Z). (E) Sequences of ten mammalian CDK4 proteins (Q9XTR1, Q7K306, Q91727, P30285, P35426, G1T3E1, P11802, H2QZZ4, P79432, G3T692) were collected from the Uniprot database. The lysine residues of CDK4 are evolutionarily stable among mammals. Multiple sequence alignment was accomplished by Clustal Omega and the figure was generated by WebLogo. (F) The ubiquitination of CDK4 and its 11 K-to-R mutants was detected in HEK-293T cells using denature immunoprecipitation assays. (G) The ubiquitination of CDK4 and its mutants K88R, K106R, K142R was detected in HEK-293T cells using denature immunoprecipitation assays. (H) The K48 only His-Ubi mutant was co-transfected with Myc-CDK4 into the HEK-293T cell line. After 48 h of culture, dIP was conducted to assess the K48-linked polyubiquitination of Myc-CDK4.

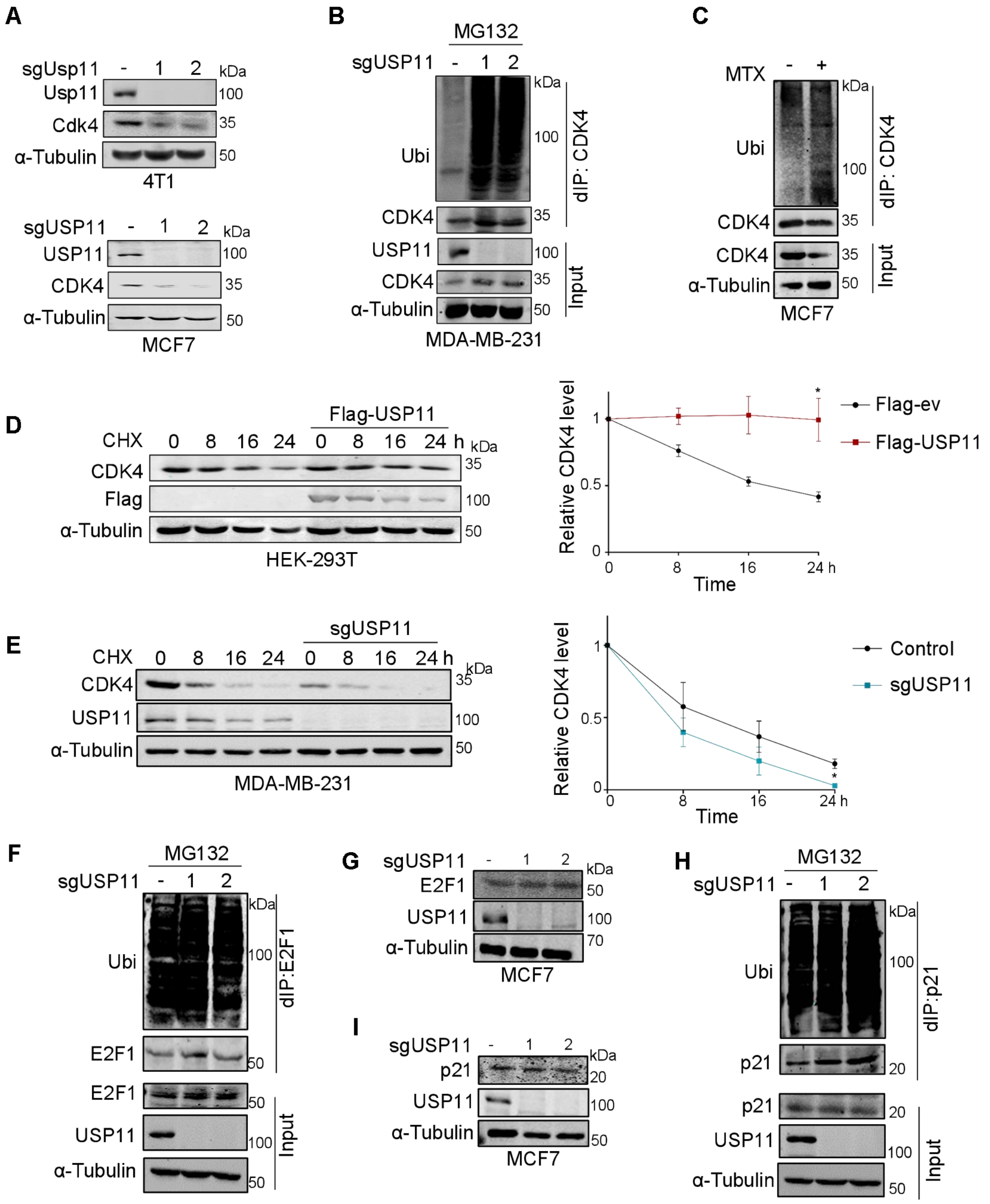

Figure S3. USP11 stabilizes CDK4 protein.

(A) USP11 knockout (KO) cell lines of 4T1 and MCF7 were successfully established using the CRISPR-Cas9 system. (B) Knockout of USP11 enhanced the ubiquitination of CDK4 in MDA-MB-231 cells. The levels of endogenous CDK4 ubiquitination in both WT and USP11 KO MDA-MB-231 cells treated with MG132 (10 μM) were assessed using dIP. (C) Inhibition of USP11 using MTX led to an increase in the ubiquitination of endogenous CDK4 in MCF7 cells. (D) The influence of USP11 overexpression on the half-life of CDK4 protein was examined in HEK-293T cells following treatment with 50 μg/mL cycloheximide (CHX). The relative quantity of CDK4 protein was determined and normalized against α-Tubulin levels. (E) The effect of USP11 KO on the half-life of CDK4 protein was assessed in MDA-MB-231 cells treated with CHX (50 μg/mL). The relative quantity of CDK4 protein was determined and normalized against the levels of α-Tubulin. (F) Analysis

of endogenous E2F1 ubiquitination in wild-type and USP11 knockout MCF7 cells. Cells were treated with 10 μM MG132 for 12 h before harvest. (G) Western blot analysis of E2F1 protein levels in wild-type and USP11 knockout MCF7 cells. (H) Analysis of endogenous p21 ubiquitination in wild-type and USP11 knockout MCF7 cells. Cells were treated with 10 μM MG132 for 12 h before harvest. (I) Western blot analysis of p21 protein levels in wild-type and USP11 knockout MCF7 cells.

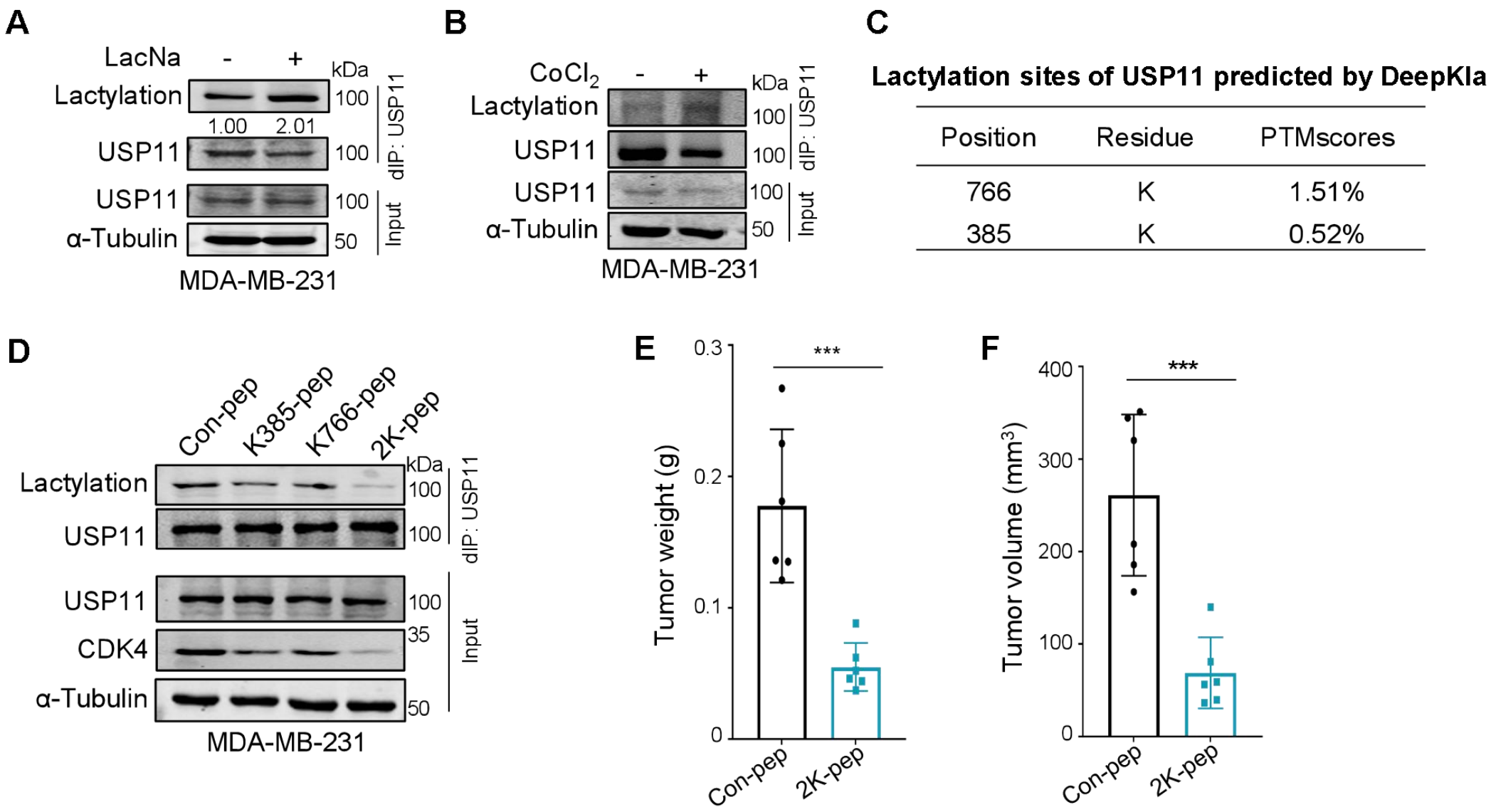

Figure S4. USP11 is a substrate of lactylation.

(A) The lactylation status of USP11 following treatment with sodium lactate was ascertained through dIP of USP11. (B) Lactylation of USP11 under hypoxic conditions induced by $CoCl_2$ was detected using dIP. $CoCl_2$ was administered at a concentration of 50 μM for 12 h to mimic hypoxia. (C) The potential lactylation sites on USP11 were predicted using DeepKla algorithm. (D) dIP showing the effect of USP11 lactylation-targeting interfering peptides (K385-pep, K766-pep, or their combination 2K-pep) on USP11 lactylation levels in MDA-MB-231 cells. A control peptide (Ctrl) was used as a negative control. (E) Quantification of tumor weights from MCF7 xenograft mice treated with control peptide or the combination of K385 and K766 interfering peptides. (F) Quantification of tumor volumes from MCF7 xenograft mice treated with control peptide or the combination of K385 and K766 interfering peptides.

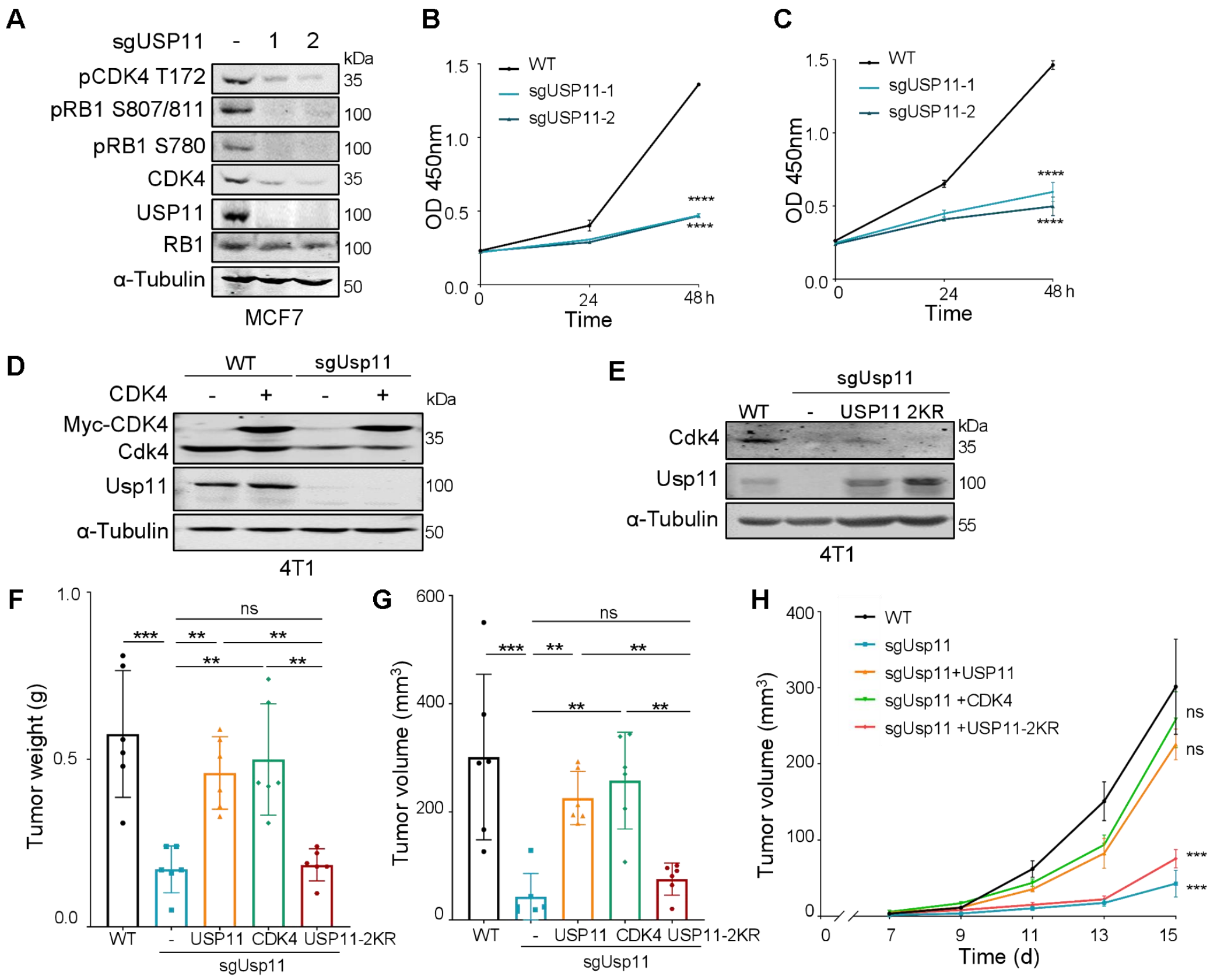

Figure S5. USP11 knockout inhibits the proliferation of breast cancer cells.

(A) The effects of USP11 depletion on the phosphorylation status of CDK4 at T172, RB1 at S780, and RB1 at S807/811 in MCF7 cells were detected using immunoblotting with specific antibodies. (B, C) The effect of USP11 KO on cell proliferation was measured in MCF7 cells (B) and MDA-MB-231 cells (C) using the CCK8 assay. Data are presented as mean ± SD (n=3). Statistical analysis was performed using one-way ANOVA. ns, not significant ($P > 0.05$). *, $P < 0.05$. **, $P < 0.01$. ***, $P < 0.001$. ****, $P < 0.0001$. (D) The expression of Myc-CDK4 was restored in WT and Usp11 KO 4T1 cells using lentivirus. (E) The impact of USP11 and its mutant USP11-2KR on CDK4 expression was assessed in Usp11 KO 4T1 cells that had been reconstituted with either USP11 or USP11-2KR. (F-H) Tumor weight (F) and tumor volume (G) from each group from Figure 4G were measured. The growth curve of tumor was shown in (H). Statistical analysis was performed using two-way ANOVA. ns, not significant ($P > 0.05$). *, $P < 0.05$. **, $P < 0.01$. ***, $P < 0.001$.

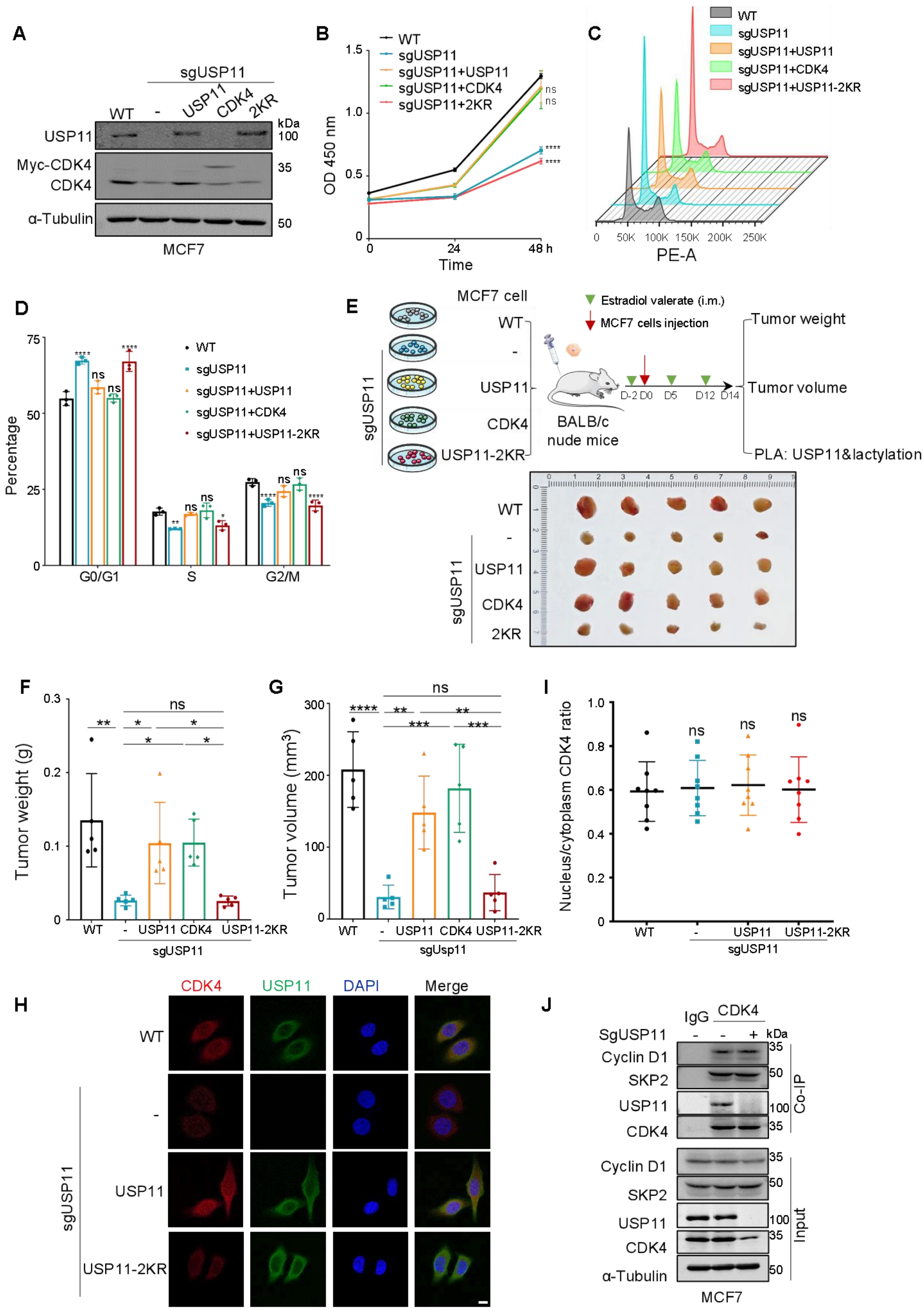

Figure S6. USP11 knockout suppresses proliferation and tumor growth of ER+ luminal breast cancer cells.

(A) The expressions of USP11, CDK4 and USP11-2KR were restored in USP11 KO MCF7 cells using lentivirus. (B) The effect of USP11 KO on cell proliferation was assessed in MCF7 cells. Additionally, the influence of rescuing USP11 WT, USP11-2KR, and CDK4 on cell proliferation was measured. (C) The impact of Usp11 KO on cell cycle was determined in MCF7 cells by flow cytometry. (D) The distribution of MCF7 cells across various phases

of the cell cycle was determined using flow cytometry. (E) WT MCF7 cells, USP11 KO MCF7 cells, and USP11 KO MCF7 cells rescued with USP11, CDK4, or USP11-2KR were separately injected into BALB/c nude mice. After 14 days, tumor weight and tumor volume were measured. Photographs of the tumors from each group (n = 5) are presented. (F, G) Tumor weight (F) and tumor volume (G) of each group from (E) were measured. Statistical analysis was performed using two-way ANOVA. ns, not significant ($P > 0.05$). *, $P < 0.05$. **, $P < 0.01$. ***, $P < 0.001$. ****, $P < 0.0001$.(H) Immunofluorescence analysis of CDK4 subcellular localization in control, USP11 knockout, USP11 KO reconstituted with wild-type USP11, and USP11 KO reconstituted with USP11-2KR MCF7 cells. CDK4 (red), USP11 (green) and nuclei (DAPI, blue) are shown. Scale bar, 5 μm. (I) Quantification of the ratio of nuclear to cytoplasmic CDK4 fluorescence intensity from the experiment shown in Fig. S6H. Statistical analysis was performed using one-way ANOVA. ns, not significant. (J) Co-IP analysis of the interaction between CDK4 and SKP2, as well as between CDK4 and Cyclin D1, in wild-type and USP11 knockout MCF7 cells.

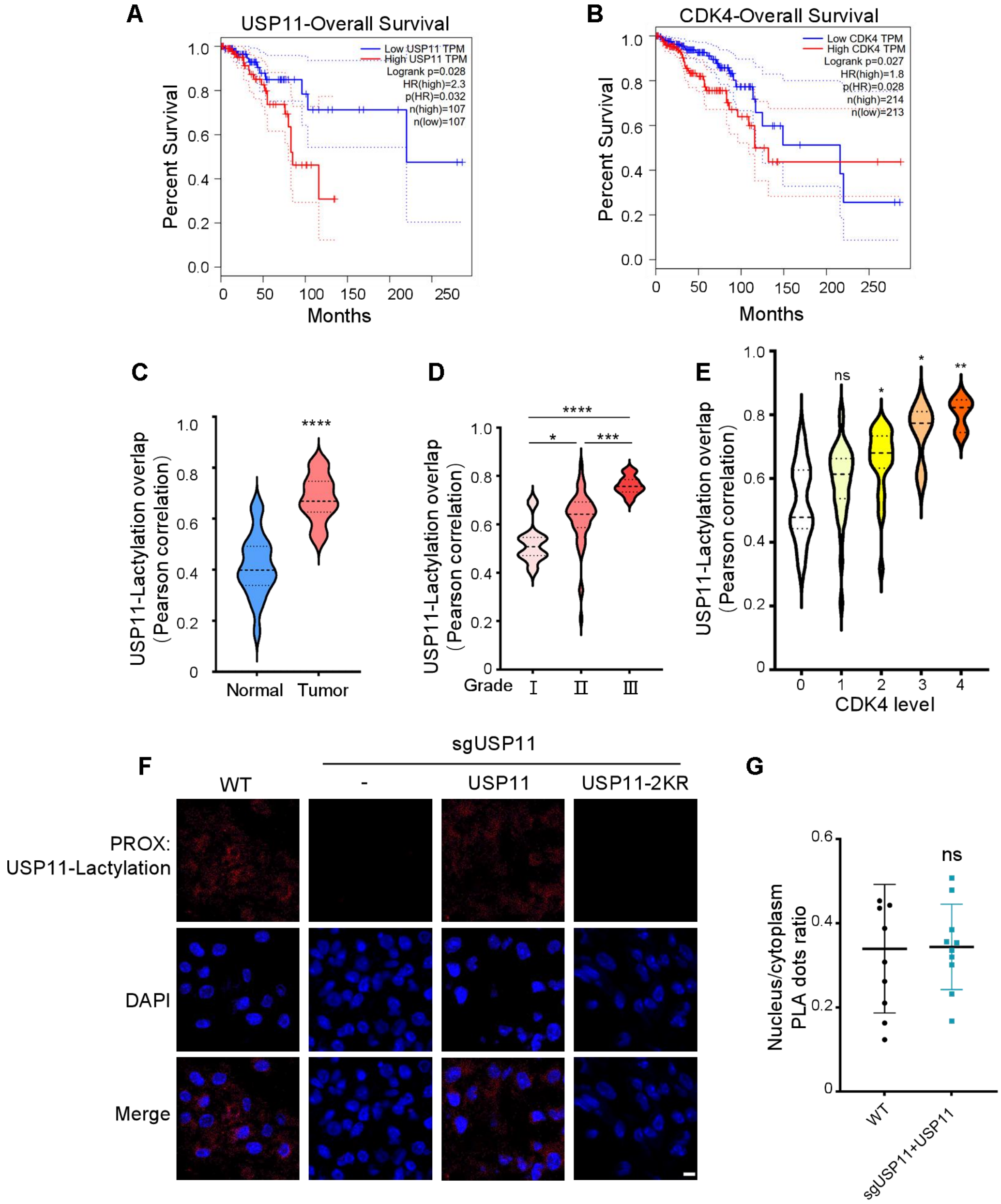

Figure S7. USP11 and its lactylation are implicated in breast cancer progression. (A) The correlation between overall survival and USP11 mRNA expression levels in breast cancer patients was examined using data from the TCGA database. Patients were classified into low (n=107) and high (n=107) USP11 expression groups based on a 10% cutoff. (B) The correlation between overall survival and CDK4 mRNA expression levels in breast cancer patients was analyzed using the TCGA database. Patients were categorized into low (n=213) and high (n=214) CDK4 expression groups based on a 20% cutoff. (C) Quantification of USP11 - lactylation colocalization in adjacent normal and breast tumor tissues (n = 20) using Pearson's correlation coefficient. Statistical significance was determined by Student's *t*-test. ****P < 0.0001. (D) Pearson's correlation coefficients of USP11 - lactylation colocalization stratified by tumor pathological grade (Grade I, II, III). Statistical significance was determined by one - way ANOVA. *P < 0.05,

***P < 0.001, ****P < 0.0001. (E) Correlation between USP11‐lactylation colocalization (Pearson's coefficient) and CDK4 protein expression levels in breast cancer tissues. Statistical analysis was performed using one‐way ANOVA. ns, not significant ($P > 0.05$). *, $P < 0.05$. **, $P < 0.01$. (F) Duolink PLA detecting endogenous USP11 lactylation in control, USP11‐knockout, USP11 KO reconstituted with wild‐type USP11, and USP11 KO reconstituted with USP11‐2KR tumors from Figure S6E. Scale bar, 5 μm (G) Quantitative analysis of the subcellular distribution of USP11 lactylation PLA signals in breast tumor tissues from (F). The ratio of PLA signals localized in the nucleus versus the cytoplasm is shown. Statistical significance was determined by Student's *t*‐test. ns, not significant ($P > 0.05$).

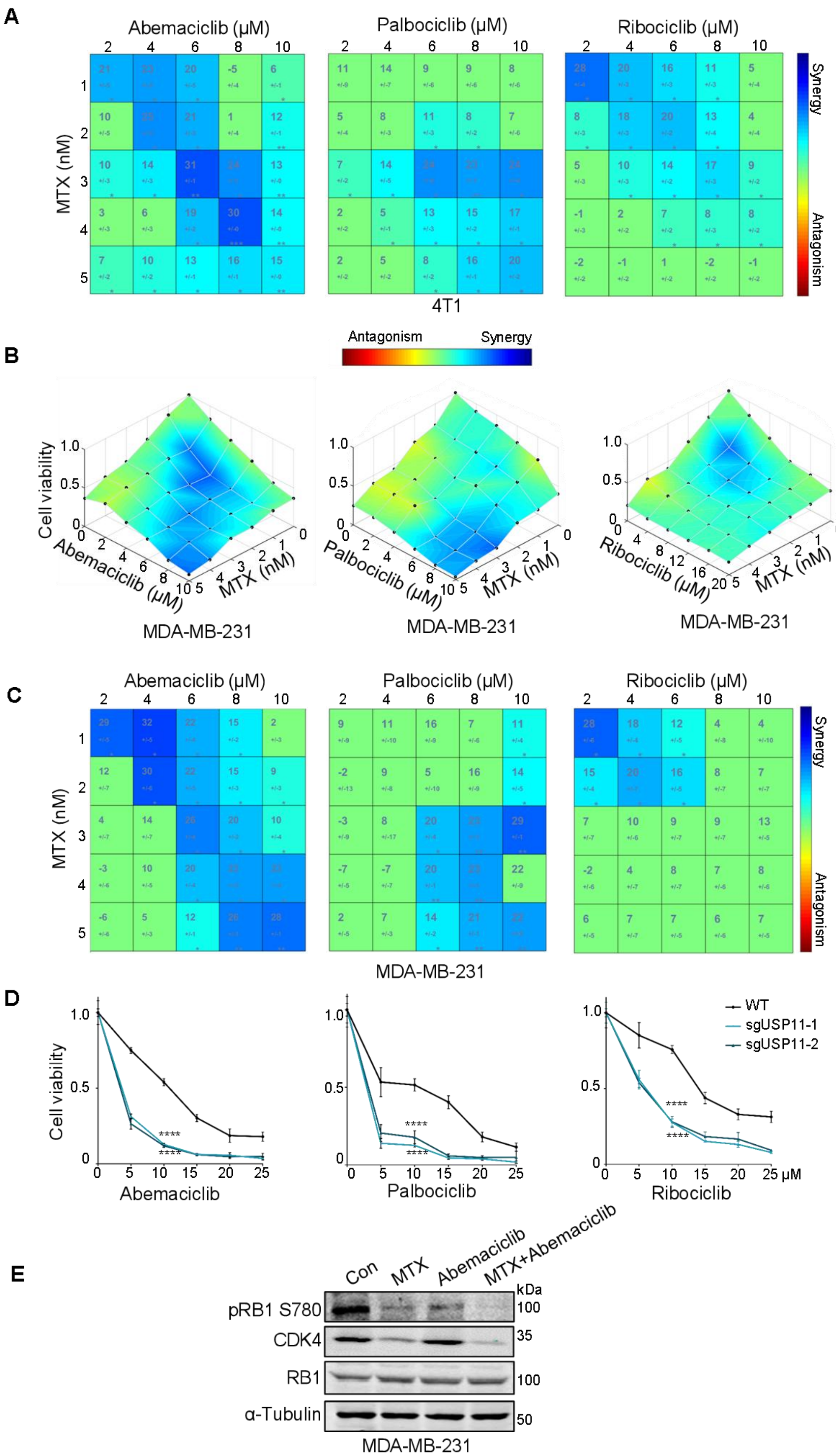

Figure S8. The combination of USP11 inhibitor and CDK4 inhibitor exhibits a synergistic effect.

(A) The quantitative HSA synergy scores for the drug combinations of USP11 inhibitor and CDK4 inhibitor in 4T1 cells. (B) The *in vitro* synergetic antitumor effects of MTX in combination with Abemaciclib, Palbociclib, or Ribociclib were evaluated across a range of concentrations using the CCK8 assay in MDA-MB-231 cells. The graphical representation of the data was generated by Combenefit software. (C) The quantitative HSA synergy scores for the drug combinations of USP11 inhibitor and CDK4 inhibitor in MDA-MB-231 cells. (D) The sensitivities of USP11 KO MDA-MB-231 cells to Abemaciclib, Palbociclib, and Ribociclib were assessed. (E) Western blot analysis of p-RB1 (S780) in MDA-MB-231 cells treated with DMSO, MTX (10 nM), Abemaciclib (2.5 μM), or their combination for 24 h.

SI References

1. Bolte S, Cordelieres FP. A guided tour into subcellular colocalization analysis in light microscopy. *J Microsc* 2006, **224**(Pt 3)**:** 213-232.
2. Tovchigrechko A, Vakser IA. GRAMM-X public web server for protein-protein docking. *Nucleic Acids Res* 2006, **34**(Web Server issue)**:** W310-314.
3. Chong LT, Pitera JW, Swope WC, Pande VS. Comparison of computational approaches for predicting the effects of missense mutations on p53 function. *J Mol Graph Model* 2009, **27**(8)**:** 978-982.
4. Di Veroli GY, Fornari C, Wang D, Mollard S, Bramhall JL, Richards FM*, et al.* Combenefit: an interactive platform for the analysis and visualization of drug combinations. *Bioinformatics* 2016, **32**(18)**:** 2866-2868.
5. Tang Z, Kang B, Li C, Chen T, Zhang Z. GEPIA2: an enhanced web server for large-scale expression profiling and interactive analysis. *Nucleic Acids Res* 2019, **47**(W1)**:** W556-W560.
